# β_2_-integrins control HIF1α activation in human neutrophils

**DOI:** 10.3389/fimmu.2024.1406967

**Published:** 2024-10-14

**Authors:** Lovis Kling, Claudia Eulenberg-Gustavus, Uwe Jerke, Anthony Rousselle, Kai-Uwe Eckardt, Adrian Schreiber, Ralph Kettritz

**Affiliations:** ^1^ Experimental and Clinical Research Center, a cooperation between the Max Delbrück Center for Molecular Medicine in the Helmholtz Association and Charité – Universitätsmedizin Berlin, Berlin, Germany; ^2^ Department of Nephrology and Medical Intensive Care, Charité – Universitätsmedizin Berlin, Corporate Member of Freie Universität Berlin and Humboldt-Universität zu Berlin, Berlin, Germany

**Keywords:** neutrophils, myeloid cells, monocytes, hypoxia-inducible factors, integrins, inflammation, adhesion, hypoxia

## Abstract

During inflammation, human neutrophils engage β_2_-integrins to migrate from the blood circulation to inflammatory sites with high cytokine but low oxygen concentrations. We tested the hypothesis that the inhibition of prolyl hydroxylase domain-containing enzymes (PHDs), cytokines, and β_2_-integrins cooperates in HIF pathway activation in neutrophils. Using either the PHD inhibitor roxadustat (ROX) (pseudohypoxia) or normobaric hypoxia to stabilize HIF, we observed HIF1α protein accumulation in adherent neutrophils. Several inflammatory mediators did not induce HIF1α protein but provided additive or even synergistic signals (e.g., GM-CSF) under pseudohypoxic and hypoxic conditions. Importantly, and in contrast to adherent neutrophils, HIF1α protein expression was not detected in strictly suspended neutrophils despite PHD enzyme inhibition and the presence of inflammatory mediators. Blocking β_2_-integrins in adherent and activating β_2_-integrins in suspension neutrophils established the indispensability of β_2_-integrins for increasing HIF1α protein. Using GM-CSF as an example, increased HIF1α mRNA transcription via JAK2-STAT3 was necessary but not sufficient for HIF1α protein upregulation. Importantly, we found that β_2_-integrins led to HIF1α mRNA translation through the phosphorylation of the essential translation initiation factors eIF4E and 4EBP1. Finally, pseudohypoxic and hypoxic conditions inducing HIF1α consistently delayed apoptosis in adherent neutrophils on fibronectin under low serum concentrations. Pharmacological HIF1α inhibition reversed delayed apoptosis, supporting the importance of this pathway for neutrophil survival under conditions mimicking extravascular sites. We describe a novel β_2_-integrin-controlled mechanism of HIF1α stabilization in human neutrophils. Conceivably, this mechanism restricts HIF1α activation in response to hypoxia and pharmacological PHD enzyme inhibitors to neutrophils migrating toward inflammatory sites.

## 1 Introduction

Neutrophils and monocytes circulate in the blood from where they migrate to inflammatory sites. Once emigrated from the vasculature, myeloid cells encounter inflammatory mediators, interact with extracellular matrix proteins employing integrins (1, 2), and are exposed to hypoxic conditions (3, 4). Several cellular pathways ensure proper functioning under these challenging conditions, including the activation of hypoxia-inducible factors (HIFs) (5).

HIFs are ubiquitously expressed heterodimeric transcription factors consisting of an isoform-specific alpha-subunit and a common beta-subunit (also termed ARNT) (6, 7). Both the alpha- and beta-subunits are continuously synthesized, but oxygen-dependent proteasomal degradation of the alpha-subunit initiated by prolyl hydroxylase domain-containing enzymes 1–3 (PHD1-3) regulates heterodimerization (8–10) and thereby the transactivating activity (11–13). Recently, HIF stabilizers entered clinical practice for renal anemia treatment (14), including roxadustat (ROX) (15). These drugs inhibit PHD activity independent of oxygen tension, hence inducing a condition referred to as pseudohypoxia (16), leading to HIF pathway activation.

The activation of the HIF pathway and transcriptional upregulation of HIF target genes enables cellular metabolism and functioning of several cell types, including myeloid cells (17, 18). However, most data supporting a role of HIFs in myeloid cells were derived from animal models (19–23) and isolated murine cells (24, 25). In contrast, mechanistic HIF data obtained from human myeloid cells (18), particularly neutrophils are scarce (26). A recent study in COVID-19 patients combined peripheral blood single-cell RNA sequencing with single-cell proteomics. Despite hypoxia, the investigators observed only a weak HIF1α transcriptomic signature in one of eight identified blood neutrophil subclusters but this finding did not translate into detectable HIF protein (27). By contrast, transcriptomic sequencing of human nasopharyngeal swab samples (28) and broncho-alveolar fluid neutrophils (29) in COVID-19 showed upregulation of the HIF1α downstream target gene *VEGFA* in neutrophils that had transmigrated from the vasculature into the infected mucosa, suggesting that the HIF transcription factor had been assembled (28, 29). Inspired by these observations, we hypothesized that myeloid cells, either resting or exposed to inflammatory mediators, activate HIFs and that this effect is controlled by β_2_-integrin engagement, in addition to PHD inhibition. We achieved PHD inhibition either pharmacologically using ROX (pseudohypoxia) or by exposure to low oxygen concentration mimicking the inflammatory site microenvironment. Our data establish a novel HIF1α activation mechanism that is under the control of β_2_-integrins with relevance for neutrophil survival at extravascular inflammatory sites. Conceivably, this mechanism could be relevant to neutrophil-mediated diseases, including inflammatory bowel disease, pyelonephritis, abscesses, and autoimmune vasculitis.

## 2 Materials and methods

### 2.1 Preparation of neutrophils and monocytes from human blood samples

The study was approved by the local ethic committee (EA4/025/18), and healthy blood donors provided written informed consent. Neutrophils and monocytes were isolated from heparinized venous whole-blood samples using density-gradient centrifugation as described previously (30, 31). In brief, 1% dextran was added for red blood cell sedimentation followed by Ficoll-Hypaque density gradient centrifugation of the resulting supernatant. Monocytes were isolated from the interphase and remaining erythrocytes in the pellet containing neutrophils were hypotonically lysed for 15 s before normal osmolality was achieved through the addition of 3.6% sodium chloride solution. Neutrophils were centrifuged (10 min at 1,050 rpm), and isolated monocytes and neutrophils were resuspended in HBSS^++^ (Gibco, Waltham, USA) and counted using a Beckman Coulter system with a purity of >95%.

### 2.2 Reagents

Roxadustat (TargetMol, Wellesley Hills, USA), YC1, LPS serotype O111:B4, Actinomycin D, rapamycin, 4EGI-1, and cycloheximide (Merck, Darmstadt, Germany) were diluted in DMSO. Human recombinant TNFα, GM-CSF, IL6, IL8 (Bio-Techne GmbH, Wiesbaden, Germany), and G-CSF (PeproTech, Waltham, USA) were diluted in PBS (Gibco, Waltham, USA) with 0.1% bovine serum albumin (BSA, Merck). fMLP (Merck) was diluted in water. Monoclonal antibodies blocking integrin activation were mouse anti-human IgG CD11b clone 2LPM19c (#sc-20050, RRID:AB_626883, Santa Cruz Biotechnology, Heidelberg, Germany) and mouse anti-human IgG CD18 clone 7E4 (#IM1567, RRID:AB_131640, Beckman Coulter, Krefeld, Germany). The integrin activating antibodies were mouse anti-human IgG CD11b clone Bear1 (#IM0190, RRID:AB_3095685, Beckman Coulter) and mouse anti-human IgG CD18 clone MEM-148 (#MCA2086XZ, RRID:AB_323901, Bio-Rad, Feldkirchen, Germany). The murine IgG_1_ isotype control was from Sigma-Aldrich (#M5284, RRID:AB_1163685, Merck). The blocking PECAM-1 antibody was mouse monoclonal anti-human IgG CD31 clone Gi18 (#ALX-805-003A-C100, RRID:AB_2051038, Enzo Life Sciences, Lörrach, Germany). The exclusion of reagent cytotoxic side effects is shown in **Supplementary Figure 1**.

### 2.3 Electrophoresis and immunoblotting

For the preparation of whole-cell lysates, 1.25×10^6^ monocytes or 2.5×10^6^ neutrophils were resuspended in 50 µl of sonication buffer (150 mM Tris, pH 7.8, 1.5 mM EDTA, and 10 mM KCl) supplemented with protease inhibitors (Protease Inhibitor Cocktail III, 2 mM PMSF, 1 mM Na_3_VO_4_, 0.5 mM DTT, 2 mM levamisole, cOmplete™ 25×), sonicated in a Bioruptor® Plus sonication device (Diagenode, Seraing, Belgium) for 10 min, and centrifuged at 13,000 g for 10 min at 4°C. Whole-cell lysates in the supernatants were collected and boiled with the appropriate volume of reducing loading buffer (4× ROTI® LOAD, Carl Roth GmbH+Co.KG, Karlsruhe, Germany) at 95°C for 5 min after photometric protein quantification using ROTI® Nanoquant 5× reagent (Carl Roth GmbH+Co.KG) and a VERSAmax™ microplate reader (Molecular Devices, Sunnyvale, USA).

For HIF1α and HIF2α, 10 µg (monocytes) or 30 µg of protein (neutrophils) per sample were loaded onto 8% SDS-polyacrylamide gels and transferred to PVDF membranes (pore size 0.2 µM, Thermo Scientific, Waltham, USA). For other proteins, we used 15% SDS-polyacrylamide gels for gel electrophoresis. Membranes were blocked with 5% skimmed milk for 1 h before overnight incubation with the appropriate dilution of primary antibody in 5% BSA-containing buffer at 4°C. The following primary antibodies were used: rabbit polyclonal anti-human IgG HIF1α (1:500, #3716, RRID:AB_2116962, Cell Signaling Technologies, Leiden, The Netherlands), monoclonal mouse anti-human IgG HIF2α clone ep190b (1:500, #NB100-132, RRID:AB_10000898, Novus Biologicals, Wiesbaden, Germany), polyclonal rabbit anti-human IgG HIF2α (1:500, #NB100-122, RRID:AB_535687, Novus Biologicals), polyclonal rabbit anti-human IgG HIF2α (1:500, # PA1-16510, RRID:AB_2098236, Thermo Fisher Scientific), monoclonal rabbit anti-human IgG β-actin clone 13E5 (1:2000, #4970, RRID:AB_2223172), monoclonal rabbit anti-human IgG phospho-STAT3 (Tyr705) clone D3A7 (1:1000, #9145, RRID:AB_2491009), monoclonal mouse anti-human IgG STAT3 clone 124H6 (1:1000, #9139, RRID:AB_331757), polyclonal rabbit anti-human IgG phospho-eIF4E (Ser209) (1:1000, #9741, RRID:AB_331677), monoclonal rabbit anti-human IgG phospho-4EBP1 (Ser65) clone D9G1Q (1:1000, #9451, RRID:AB_330947), monoclonal rabbit anti-human IgG mTOR clone 7C10 (1:1000, #2983, RRID:AB_2105622), and polyclonal rabbit anti-human IgG phospho-mTOR (Ser2448) (1:1000, #2971, RRID:AB_330970, all from Cell Signaling Technologies). The secondary HRP-conjugated antibodies were diluted in 5% skimmed milk 1:1000-1:5000 (rabbit anti-mouse IgG from Agilent Technologies Denmark, #P0260, RRID:AB_2636929, and donkey anti-rabbit IgG from Cytiva, #NA934V, RRID:AB_772206). Chemiluminescence was detected on a VWR ECL reader using SuperSignal™ West Dura Extended Duration Substrate (Thermo Fisher Scientific). All bands presented were at the predicted molecular weight for the protein of interest. Densitometric analysis was performed using ImageJ (RRID: SCR_003070). Fold changes of protein abundance were calculated from optical density ratios of the target protein and the loading control was normalized to the ROX-treated sample (or as specified in the figure legends).

### 2.4 Coating of tissue culture wells

PolyHema (poly 2-hydroxyl-ethyl methacrylate, Merck) was dissolved in 95% ethanol at 80 mg/ml and 500 µl per well was pipetted on a 12-well tissue culture plate (TPP Techno Plastic Products AG, Trasadingen, Switzerland) and left to dry overnight. Fibronectin (Roche, Mannheim, Germany) was dissolved at 20 µg/ml in HBSS^++^ before the wells were coated for 1 h at room temperature.

### 2.5 Neutrophil migration assay

Fibronectin-coated sterile transwells (3 µm pore diameter, Sarstedt, Germany) were inserted into a tissue culture plate containing HBSS^++^, chemoattractant, and ROX in the appropriate experimental conditions. Neutrophils were pipetted in the transwell and were allowed to migrate to the lower well at 37°C with 5% CO_2_. After 4 h, migrated neutrophils were either collected for protein extraction (see immunoblotting) or lysed using 0.5% Triton X-100 for the measurement of MPO activity using a 2,2′-Azino-bis(3-ethylbenzothiazoline-6-sulfonic acid)-based (Merck) colorimetric assay on a VERSAmax™ microplate reader (Molecular Devices).

### 2.6 Neutrophil apoptosis

Neutrophils were resuspended in Roswell Park Memorial Institute (RPMI) medium (Gibco) supplemented with autologous serum and cultured for 4 h/20 h at 37°C with 5% CO_2_ with 2.5×10^6^ cells per well. Neutrophil adhesion was induced by fibronectin-coated wells. Apoptotic neutrophils were washed, transferred to Annexin binding buffer (BD Biosciences, Heidelberg, Germany), and co-stained with Annexin V-APC (BD Biosciences) and 7-AAD (Merck). FACS analysis was performed immediately after staining on a BD CANTO II cytometer. FACS data were analyzed on FlowJo version 7 (Treestar, USA).

### 2.7 Quantitative PCR

For qPCR, RNA was transcribed into cDNA after deoxyribonuclease I-treatment (Qiagen, Venlo, The Netherlands) using hexanucleotide primers and the RevertAid First Strand cDNA Synthesis Kit following the manufacturer’s protocol (Thermo Fisher Scientific). qPCR was performed with the Fast SYBR Green Master Mix or the TaqMan Fast Universal PCR Master Mix (Applied Biosystems, Waltham, USA) and run on a QuantStudio plus machine (Applied Biosystems). Primers for qPCR were designed with Primer 3 and were as follows: HIF1α (forward, 5′-CATAAAGTCTGCAACATG GAAGGT-3’; reverse, 5′-ATTTGATGGGTGAGGAATGGGTT-3′), 18S (forward, 5′-ACATCCAAGGAAGGCAGCAG-3′; reverse, 5′-TTTTCGTCACTACCTCCCCG-3′, 5′-6-FAM-CGCGCAAATTACCCACTCCCGAC-TAMRA-3′), and VEGFA (forward, 5′-GAGGAGGGCAGA ATCATCAC-3′; reverse, 5′-ACACAGGATGGCTTGAAGATG-3′). Quantitation was performed using the ΔΔCT-method using the ROX-treated sample as the reference sample.

### 2.8 Statistics

Results are given as means ±standard deviation. Statistical comparisons were made using the ratio-paired *t*-test for 2 groups and one-way ANOVA for experimental groups of >2 using GraphPad Prism9 software. Multiple testing correction was performed using Šidák’s method. Differences were considered significant at *p*<0.05 (*), *p*<0.01 (**), *p*<0.001 (***), or *p*<0.0001 (****).

## 3 Results

### 3.1 Inflammatory mediators increase HIF1α protein marginally but some act synergistically with pseudohypoxia in upregulating HIF1α protein in neutrophils

**Figure 1:**
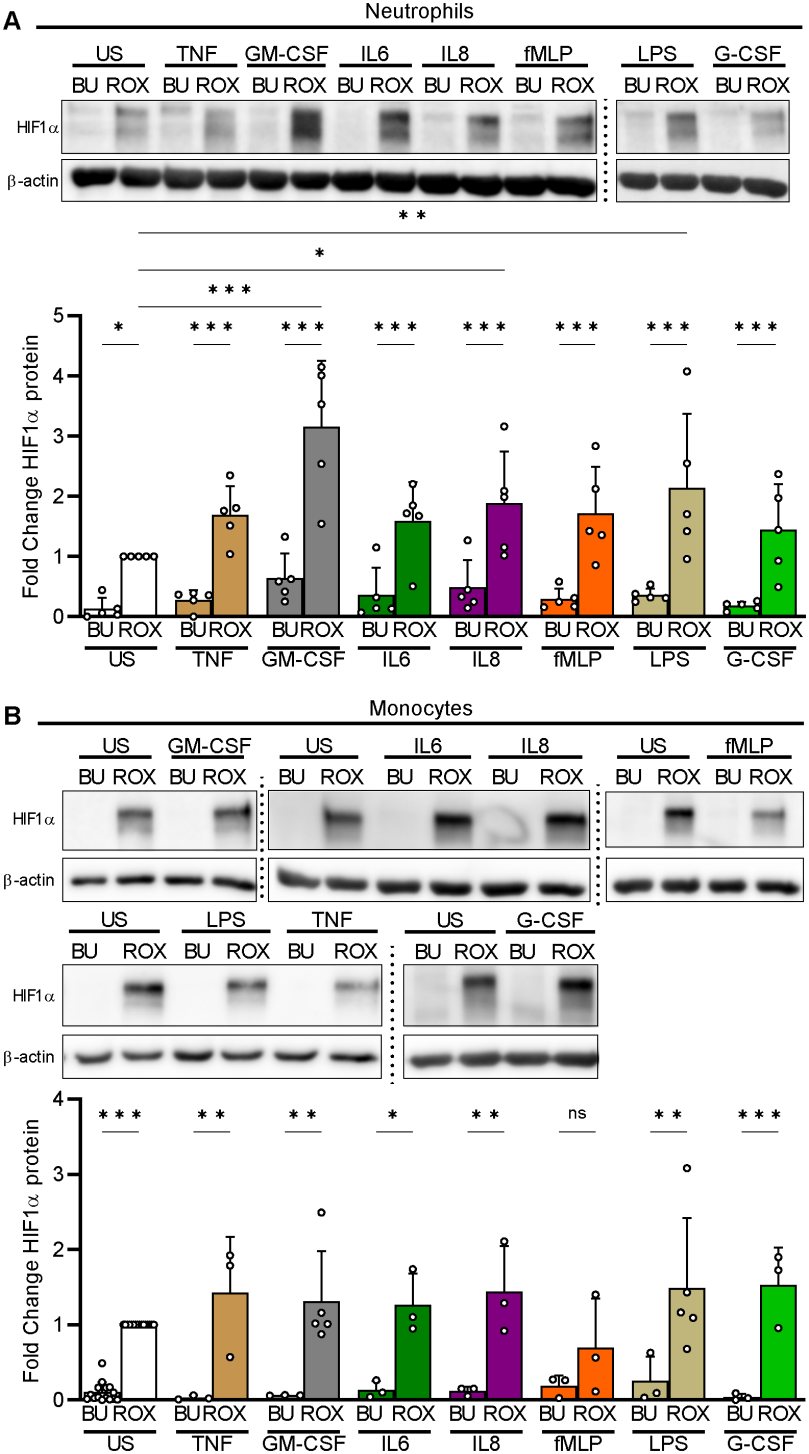
HIF1α protein expression under pseudohypoxia and inflammatory conditions in human myeloid cells. **(A)** Freshly isolated human neutrophils were left unstimulated (US) or treated in parallel with the indicated inflammatory mediators (TNFα 2 ng/ml, GM-CSF 20 ng/ml, IL6 20 ng/ml, IL8 100 nM, fMLP 10 nM, LPS 1 µg/ml, and G-CSF 100 ng/ml) in the presence of buffer (BU) or 15 µM ROX for 4 h in Eppendorf tubes (n=5). HIF1α was detected using immunoblotting with a specific antibody followed by an assessment of the optical densities (OD) of the bands. The ROX condition was set as a reference for the statistical analysis of the HIF1α protein fold change. A representative experiment is shown. **(B)** Freshly isolated human monocytes were incubated in the presence of BU or ROX with the indicated stimuli for 4 h in tubes (n=3–5). A representative experiment is depicted. Statistical analysis was performed by repeated measure one-way ANOVA (neutrophils, **A**) or mixed-effects analysis (monocytes, **B**) with Šidák’s multiple comparison test of the conditions investigated in parallel. ns, not significant. p<0.05 (*), p<0.01 (**), p<0.001 (***).

We first assessed HIF1α in neutrophils both resting and exposed to a variety of inflammatory mediators during culture in test tubes. Under normoxia, we did not detect HIF1α protein in the former and observed a marginal HIF1α signal at most in the latter (**Figure 1A**). Under pseudohypoxia with ROX-dependent PHD inhibition, HIF1α protein significantly increased in resting neutrophils. Moreover, IL8 acted additively, and GM-CSF and LPS even synergistically to pseudohypoxia in HIF1α protein upregulation. We selected 15 µM of ROX because reported blood concentrations in patients range from 2.8–28 µM (32–34) and determined a 4 h incubation period based on a time course study (**Supplementary Figure 2A**).

When we investigated isolated human blood monocytes in test tubes, we found that pseudohypoxia, similar to neutrophils, reliably led to HIF1α protein accumulation in resting and stimulated cells but, in contrast to neutrophils, none of the inflammatory mediators showed additive or synergistic effects with ROX (**Figure 1B**).

We did not detect HIF2α protein in resting and stimulated neutrophils as well as monocytes under pseudohypoxia using three different antibodies that detected HIF2α in appropriate control lysates (**Supplementary Figures 2B, C**).

Thus, inflammatory mediators were weak myeloid cell HIF1α activators at most but acted additively or even synergistically with PHD inhibition in human neutrophils. Based on these findings, we focused on HIF1α regulation in neutrophils and selected GM-CSF as a synergistic inflammatory stimulus for further experiments.

### 3.2 β_2_-integrin-mediated adhesion is indispensable for neutrophil HIF1α protein induction

**Figure 2:**
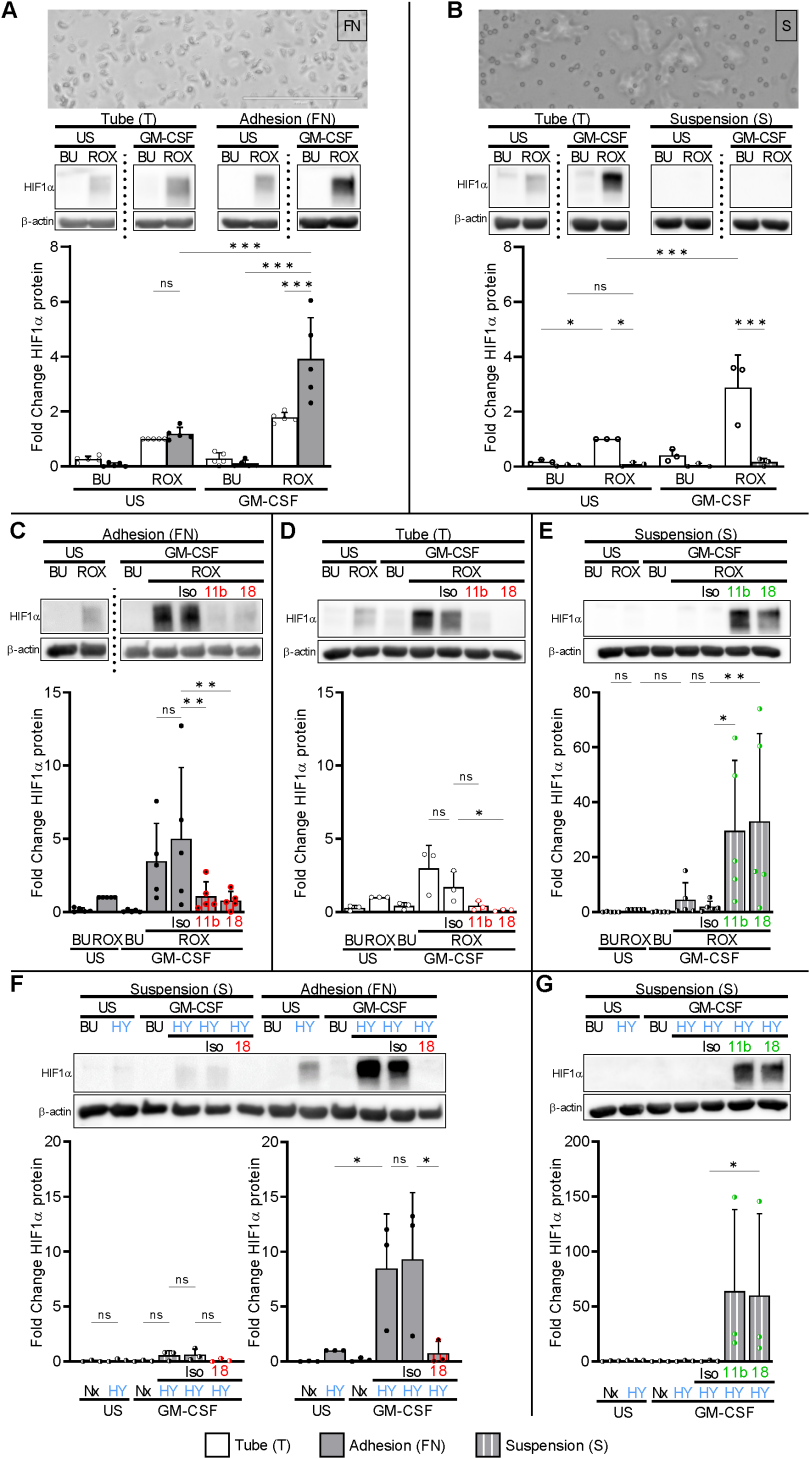
Integrin-dependent HIF1α protein expression in human neutrophils. **(A)** Freshly isolated human neutrophils were incubated in fibronectin (FN)-coated wells (gray bars), either unstimulated (US) or treated with 20 ng/ml GM-CSF in the absence (buffer, BU) or presence of 15 µM ROX for 4 h. Parallel stimulation was performed in Eppendorf tubes (T, white bars) (n=5). Phase contrast microscopy on an Invitrogen™ EVOS™ Digital Fluorescence Microscope (with an integrated Sony ICX445 monochrome CCD camera and software) was performed after 60 min, indicating adhesion and spreading on FN (original magnification 20×, numerical aperture 0.4, bar represents 200 µm). The lower picture section is depicted. HIF1α was detected by immunoblotting. HIF1α protein fold change was normalized to the ROX condition in tubes (T). **(B)** Freshly isolated human neutrophils were cultured in suspension on PolyHema-coated wells (S, hatched bars) and in tubes (T) in parallel (n=3). Treatment and analyses were performed as in **(A)**. Note that cells on PolyHema-coated wells were mostly isolated from each other and did not spread. **(C)** 20 µg blocking mAbs to CD11b (11b) and CD18 (18) or isotype control (Iso) was added to neutrophils on FN (n=5) and in **(D)** neutrophils stimulated in Eppendorf tubes (T) (n=3). The ROX condition served as a control for the HIF1α fold change calculations. **(E)** 10 µg activating mAbs to CD11b (11b), CD18 (18), or isotype control (Iso), respectively, were added to suspended (S) neutrophils (n=5). The ROX condition served as a control for the HIF1α fold change calculations. **(F)** Suspended (S) neutrophils and neutrophils on FN were treated with 20 ng/ml GM-CSF or untreated (US) under normoxia (21% O_2_, Nx) or normobaric hypoxia (1% O_2_, HY) for 4 h (n=3). When indicated, cells were preincubated with 20 µg of isotype control (Iso) or blocking mAb to CD18 (18). **(G)** Suspended (S) neutrophils were incubated with 20 ng/ml GM-CSF or untreated (US) under normoxia (21% O_2_, Nx) or normobaric hypoxia (1% O_2_, HY) for 4 h. Where indicated, the cells were preincubated with 10 µg of activating mAbs to CD11b (11b) and CD18 (18). The HY condition served as a control for the HIF1α fold change calculations. **(A–F)** Statistical analysis was performed by repeated measure one-way ANOVA with Šidák’s multiple comparison test. Significance was tested by the ratio-paired *t*-test in **(G)**. ns, not significant. p<0.05 (*), p<0.01 (**), p<0.001 (***).

Neutrophils encounter their inflammatory challenges either when circulating in the bloodstream or in inflamed tissues where they interact with extracellular matrix proteins. We tested the hypothesis that adhesion provides an essential factor for HIF1α protein induction limiting HIF1α effects to emigrating neutrophils. We applied two distinct conditions, namely neutrophil incubation on fibronectin (FN)-coated plates to promote extracellular matrix interactions, and PolyHema-coated plates (S) to prevent adhesion and reduce cell-cell contacts (35) mimicking bloodstream neutrophils. We confirmed that the incubation of GM-CSF-treated neutrophils on FN resulted in cell adhesion and spreading that was not observed on PolyHema (**Figures 2A, B**). We then compared these two biologically relevant conditions with our initially used assays in test tubes (T) that explicitly allow for plastic and cell-cell contact. Culturing neutrophils on FN did not result in HIF1α protein per se neither in resting nor in GM-CSF-treated cells. However, we detected HIF1α protein in resting and, fourfold stronger, in GM-CSF-treated neutrophils on FN under pseudohypoxia. Notably, the synergistic ROX/GM-CSF HIF1α effect was even more pronounced on FN than with tubes (T). In sharp contrast, HIF1α protein was not induced by pseudohypoxia under stringent suspension conditions on PolyHema (S), neither in resting nor in GM-CSF-stimulated neutrophils (**Figure 2B**). Based on these findings, we explored the role of β_2_-integrins in HIF1α induction. Blocking antibodies to CD11b or CD18 prevented HIF1α induction in GM-CSF-stimulated neutrophils incubated under pseudohypoxic conditions in tubes (T) and on FN (**Figures 2C, D**). Conversely, activating β_2_-integrin antibodies enabled HIF1α protein expression in ROX/GM-CSF-treated suspension neutrophils on PolyHema (S, **Figure 2E**). Because *VEGFA* is a target gene for the HIF1α transcription factor, we validated the β_2_-integrin dependent HIF1α pathway activation by measuring VEGFA mRNA. We observed that transcription of the HIF1α target gene *VEGFA* was only upregulated in ROX- and GM-CSF/ROX-treated adherent neutrophils on FN, whereas no significant regulation was found in suspended neutrophils on PolyHema that lacked integrin-elicited HIF1α protein expression (**Supplementary Figure 3**).

We next investigated whether normobaric hypoxia produced similar results as pharmacological PHD inhibition. Culturing neutrophils in 1% O_2_ on FN, but not in normoxia (21% O_2_) on FN, induced HIF1α protein in resting neutrophils and approximately 8.5-fold more in GM-CSF-treated neutrophils. Similar to pseudohypoxia, HIF1α protein in GM-CSF-treated hypoxia-exposed neutrophils on FN was prevented by blocking antibodies to β_2_-integrins, which was not observed in suspension neutrophils cultured on PolyHema (S), and was strongly induced by activating β_2_-integrins on PolyHema (**Figures 2F, G**). Our data establish an indispensable role for β_2_-integrins in neutrophil HIF1α induction under both pharmacological pseudohypoxic and normobaric hypoxic conditions.

### 3.3 Neutrophils migrating toward pseudohypoxia increase HIF1α protein with a synergistic GM-CSF effect

**Figure 3:**
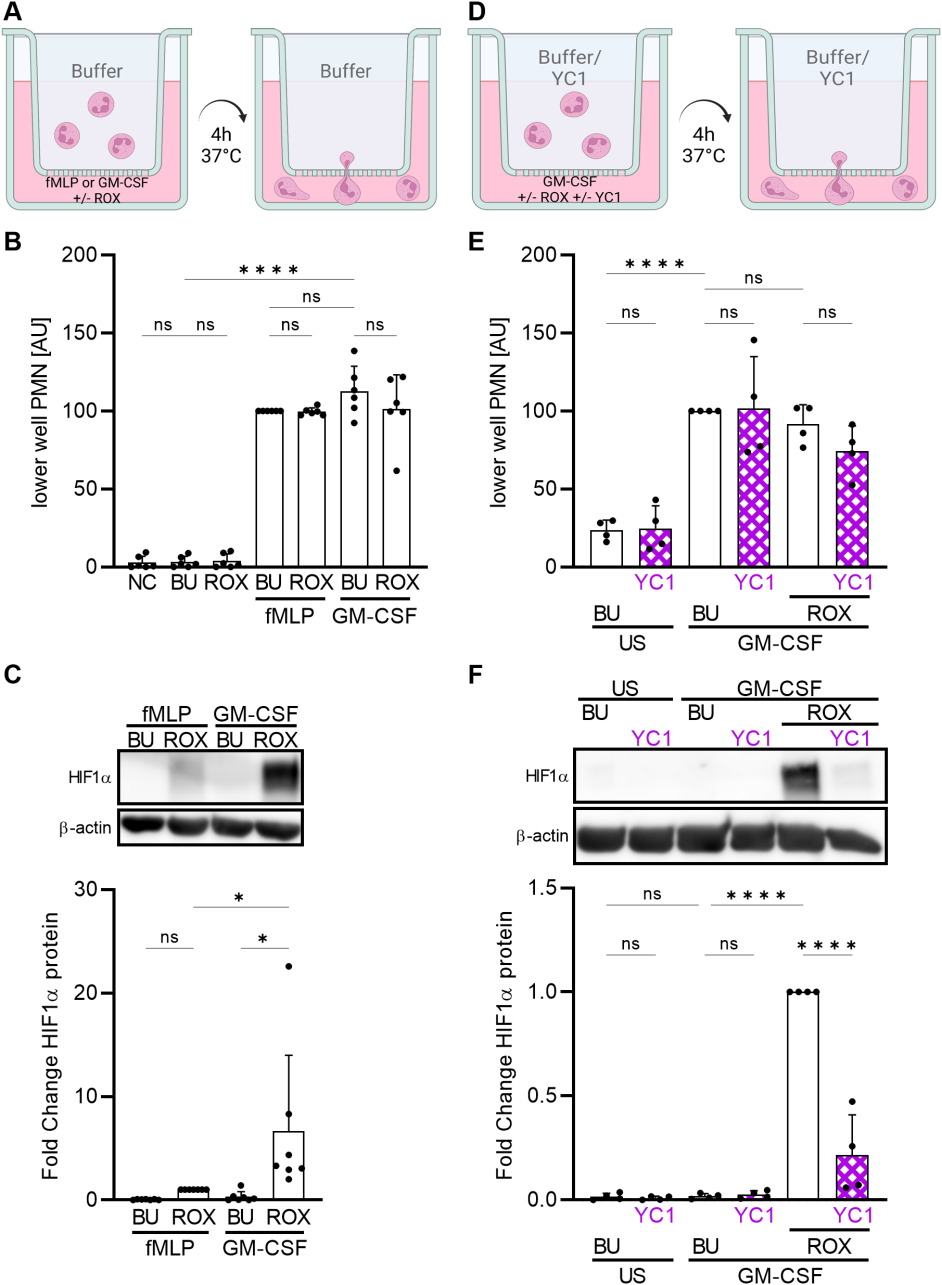
Neutrophil migration toward ROX induces HIF1α protein expression that is synergistically increased by GM-CSF. **(A)** A schematic of the experimental setting is depicted (created with biorender.com). Neutrophil migration across 3 µM FN-coated transwells and HIF1α protein was studied after 4 h at 37°C. The lower well was prepared with HBSS^++^ (NC), buffer (BU), 15 µM ROX, 10 nM fMLP, or 20 ng/ml GM-CSF or combinations thereof as indicated. **(B)** Migrated neutrophils in the lower well were quantified using a colorimetric MPO assay (n=6). Arbitrary units (AU) were calculated based on the results of the fMLP+BU condition in each experiment. **(C)** Migrated lower well neutrophils were assessed for HIF1α by immunoblotting (n=6). A representative blot and the corresponding statistics are shown. **(D)** Neutrophils were pre-treated with 10 µM YC1 or buffer (BU) for 1 h at 37°C prior to neutrophil migration. The lower well was prepared with buffer (BU), 10 µM YC1, 20 ng/ml GM-CSF, or combinations thereof as indicated. **(E)** Migrated neutrophils in the lower well were quantified using a colorimetric MPO assay (n=4). Arbitrary units (AU) were calculated based on the results of the GM-CSF+BU condition in each experiment. **(F)** Migrated lower well neutrophils were assessed for HIF1α by immunoblotting (n=4). A representative blot and the corresponding statistics are shown. **(B, C, E, F)** Statistical analysis was performed by repeated measure one-way ANOVA with Šidák’s multiple comparison test. ns, not significant. p<0.05 (*), p<0.0001 (****).

During emigration from the blood into hypoxic inflammatory sites, neutrophils move against a chemotactic gradient and interact with extracellular matrix proteins in a β_2_-integrin-dependent manner. To mimic these conditions, we performed a migration assay using FN-coated transwells and two chemoattractants (**Figure 3A**), namely GM-CSF that had, and fMLP that did not have, a synergistic HIF1α-increasing effect with ROX (**Figure 1A**). Either chemoattractant was pipetted into the lower well together with buffer control or ROX to mimic hypoxia at the inflammatory site. Neutrophil migration toward the two chemoattractants was similar and was not affected by HIF1α activation with ROX (**Figure 3B**). However, neutrophils from the lower well that had migrated towards GM-CSF/ROX increased HIF1α significantly more than those that had migrated toward fMLP/ROX (**Figure 3C**). In addition, HIF inhibition with YC1 (**Figure 3D**) also did not affect neutrophil transmigration (**Figure 3E**) but abrogated HIF1α protein accumulation in transmigrated neutrophils (**Figure 3F**). These data further support the notion that HIF1α is strongly induced in neutrophils migrating into inflammatory sites where hypoxia and specific cytokines synergistically increase HIF1α. Next, we investigated mechanisms by which GM-CSF and β_2_-integrins cooperate in HIF1α protein induction under pseudohypoxia.

### 3.4 GM-CSF induces JAK2-STAT3-mediated HIF1α transcription that is necessary but not sufficient to increase HIF1α protein in adherent neutrophils under pseudohypoxia

**Figure 4:**
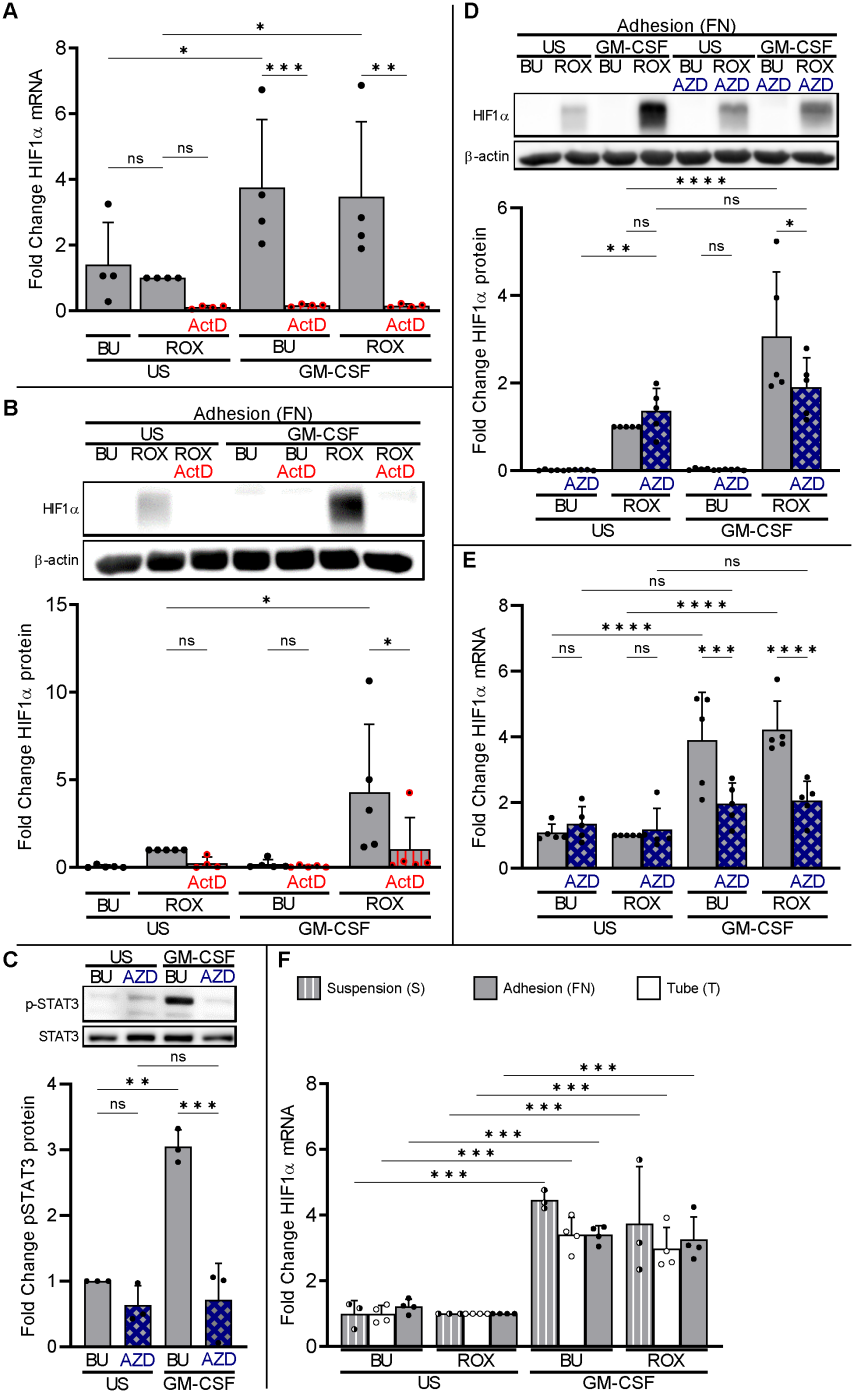
Neutrophil HIF1α transcription is necessary but not sufficient for HIF1α protein expression. **(A)** Total RNA was isolated from neutrophils cultured in fibronectin-coated wells (FN, gray bars) after 4 h incubation with 20 ng/ml GM-CSF or without (US), buffer (BU), 15 µM ROX, and 5 µg/ml Actinomycin D (ActD), as indicated. Samples were subjected to HIF1α qPCR (n=4). **(B)** Neutrophils treated as in **(A)** were prepared for HIF1α immunoblotting. A representative blot and the corresponding statistics of five experiments are shown. **(C)** Neutrophils cultured on FN were treated with GM-CSF or untreated (US) for 5 min. Samples were assessed by immunoblotting for total and phosphorylated STAT3 (n=3). Pre-incubation with 1 µM JAK2 inhibitor AZD1480 (AZD) for 30 min on ice was used to suppress GM-CSF-induced STAT3 phosphorylation. A representative blot and the corresponding statistics are shown. **(D, E)** Neutrophils cultured on FN were stimulated with 20 ng/ml GM-CSF or unstimulated (US) without (BU) or with 15 µM ROX for 4 h in the absence or presence of 1 µM AZD1480 (n=5). Protein and total RNA were isolated to detect **(D)** HIF1α protein and **(E)** mRNA, respectively. A representative immunoblot and the statistics are depicted. **(F)** Total RNA was isolated for HIF1α qPCR from neutrophils cultured for 4 h in suspension (S, hatched bars), Eppendorf tubes (T, white bars), or on FN (gray bars) in the presence of the indicated stimuli (n=3–4). The ROX condition was set as reference for ΔΔCT calculations and statistical analysis. **(A–F)** Statistical analysis was performed by repeated measure one-way ANOVA with Šidák’s multiple comparison test. ns, not significant. p<0.05 (*), p<0.01 (**), p<0.001 (***), p<0.0001 (****).

We hypothesized that GM-CSF upregulates HIF1α transcription leading to increased HIF1α protein when PHDs are inhibited. To evaluate whether neutrophil HIF1α transcription contributes to neutrophil HIF1α protein expression, we employed the dsDNA intercalating agent actinomycin D (ActD) to inhibit the transcription of RNA (36). We detected constitutive HIF1α mRNA in resting neutrophils on FN that was not affected by ROX but significantly upregulated by GM-CSF (**Figure 4A**), resulting in increased HIF1α protein when PHDs were inhibited by ROX (**Figure 4B**). ActD treatment for 4 h inhibited HIF1α transcription in ROX- and GM-CSF-treated neutrophils (**Figure 4A**) and decreased HIF1α protein in GM-CSF/ROX-treated neutrophils, indicating the need for continuously active HIF1α transcription (**Figure 4B**).

We used the JAK2 inhibitor AZD1480 to suppress GM-CSF-induced STAT3 phosphorylation (**Figure 4C**). AZD1480 abrogated the synergistic GM-CSF effect on HIF1α protein in neutrophils on FN (**Figure 4D**) by inhibiting GM-CSF-enhanced HIF1α transcription (**Figure 4E**). These data indicate that GM-CSF increases HIF1α mRNA, at least in part, using the JAK2-STAT3 pathway.

We then assessed HIF1α transcription in neutrophils incubated in PolyHema, tube, and FN conditions. GM-CSF caused a similar HIF1α mRNA increase under all three conditions (**Figure 4F**) but resulted in detectable HIF1α protein only when β_2_-integrins were engaged (see **Figure 4**). In addition, blocking β_2_-integrins in neutrophils on FN did not decrease, and activating β_2_-integrins in suspended neutrophils on PolyHema (S) did not increase, HIF1α transcription (**Supplementary Figure 4**). Thus, basal and GM-CSF-upregulated HIF1α transcription are necessary but not sufficient to explain the corresponding HIF1α protein expression in adherent neutrophils under pseudohypoxia and normobaric hypoxia. Consequently, we hypothesized that GM-CSF increased HIF1α transcription but that β_2_-integrins are essential for translation to yield HIF1α protein.

### 3.5 β_2_-integrins increase essential HIF1α translational initiation factor phosphorylation, explaining the adhesion dependency of HIF1α protein

**Figure 5:**
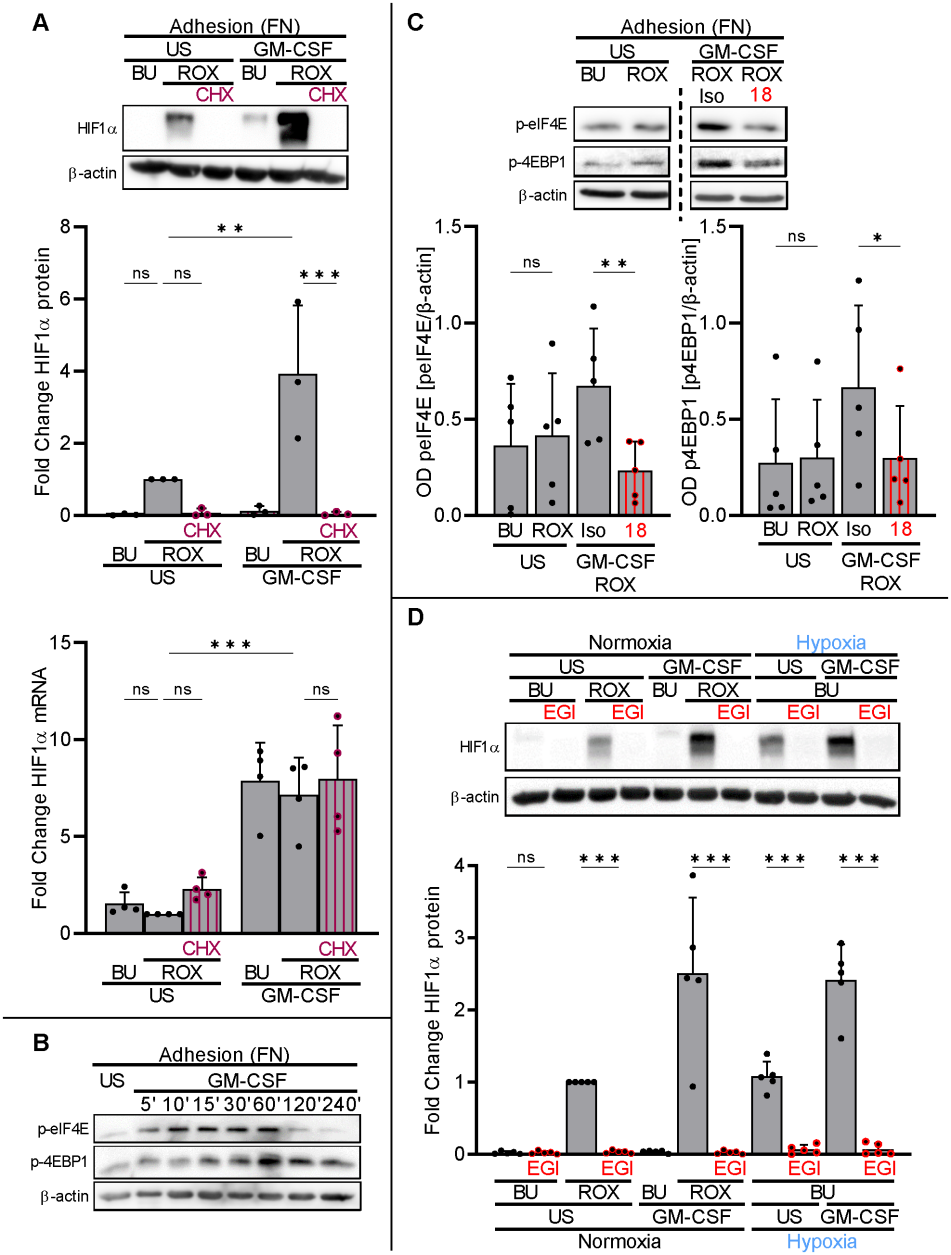
β_2_−integrins control HIF1α translation by phosphorylation of translational initiation factors. **(A)** Neutrophils cultured on FN were treated with or without (US) 20 ng/ml GM-CSF, buffer (BU), 15 µM ROX, and 2.5 µg/ml cycloheximide (CHX) as indicated. After 4 h, protein and total RNA were isolated from the samples. A representative HIF1α immunoblot (n=3) and results from HIF1α qPCR (n=4) are shown. The ROX sample was set as a reference for statistical analysis. **(B)** Neutrophils on FN were treated with BU or 20 ng/ml GM-CSF for the indicated timepoints. Immunoblotting for phospho-eIF4E (Ser209) and phospho-4EBP1 (Ser65) was performed. A representative blot of two phosphorylation time courses is presented. **(C)** Neutrophils on FN were treated without (US) or with 20 ng/ml GM-CSF, BU, 15 µM ROX, isotype (Iso), and 20 µg CD18 blocking mAb (18), as indicated. After 60 min, samples were harvested and immunoblotting performed (n=5). A representative blot is presented together with the corresponding statistical analysis. **(D)** Neutrophils on FN were incubated with GM-CSF or US, BU, ROX, and 25 µM 4EGI-1 (EGI), as indicated, under normoxia (21% O_2_) or normobaric hypoxia (1% O_2_). After 4 h, samples were harvested and HIF1α immunoblotting was performed (n=5). An exemplary blot is given together with the corresponding statistics. **(A–D)** Statistical analysis was performed by repeated measure one-way ANOVA with Šidák’s multiple comparison test. ns, not significant. p<0.05 (*), p<0.01 (**), p<0.001 (***).

To assess the contribution of translation to basal and GM-CSF-upregulated HIF1α protein in neutrophils on FN, we treated neutrophils on FN with the translation elongation inhibitor cycloheximide (CHX, 2.5 µg/ml) (37). CHX prevented HIF1α protein in resting and GM-CSF-treated neutrophils on FN under pseudohypoxia without affecting HIF1α transcription (**Figure 5A**). Together with our findings on PolyHema, namely substantial HIF1α transcription without translation into the corresponding protein, we considered β_2_-integrin-controlled HIF1α translation in human neutrophils on FN.

Translation initiation factor elF4E and 4EBP1 phosphorylation (eIF4E: Ser209, 4EBP1: Ser65) and their subsequent dissociation are rate-limiting steps for 5′-cap-dependent mRNA translation (38). The dissociation of phosphorylated 4EBP1 and eIF4E can be specifically suppressed by the compound 4EGI-1 (39, 40). We found basal phosphorylation of both co-factors in neutrophils on FN that increased with GM-CSF treatment over time with a maximum at approximately 60 min (**Figure 5B**). Selecting the 60-min timepoint, GM-CSF-induced 4EBP1 and elF4E phosphorylation was significantly reduced by the blocking CD18 antibody (**Figure 5C**). Preventing eIF4E and 4EBP1 dissociation in neutrophils on FN by 25 µM of 4EGI-1 (39), HIF1α protein levels were completely abrogated in resting and GM-CSF-stimulated neutrophils under pseudohypoxia and normobaric hypoxia (**Figure 5D**).

**Figure 6:**
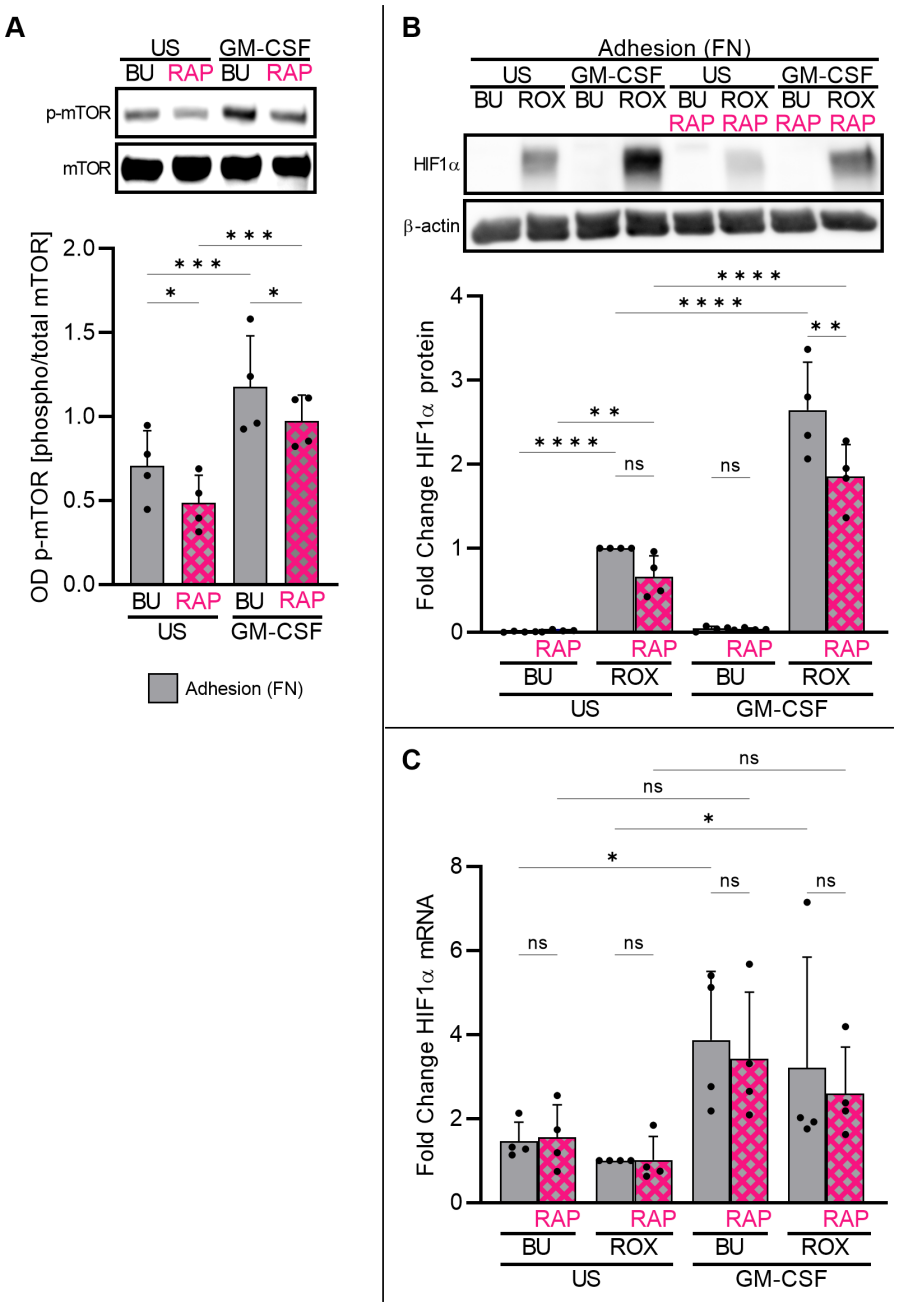
Adherent neutrophils engage the mTOR pathway in HIF1α translation. **(A)** Freshly isolated neutrophils cultured on FN were treated with GM-CSF (20 ng/ml) or left untreated (US) for 30 min. Samples were assessed by immunoblotting for total and phosphorylated mTOR at Ser2448 (n=4). Pre-incubation with 100 nM mTOR inhibitor rapamycin (RAP) for 30 min on ice suppressed mTOR phosphorylation. Representative immunoblots and the corresponding statistics are given. **(B, C)** Freshly isolated neutrophils cultured on FN were stimulated with 20 ng/ml GM-CSF or left unstimulated (US) with buffer control (BU) or 15 µM ROX for 4 h in the absence or presence of 100 nM RAP (n=4). Protein and total RNA were isolated to detect **(B)** HIF1α protein and **(C)** HIF1α mRNA, respectively. A representative immunoblot and the statistics are depicted. The ROX condition was set as a reference for ΔΔCT calculations and statistical analysis. **(A–C)** Statistical analysis was performed by repeated measure one-way ANOVA with Šidák’s multiple comparison test. ns, not significant. p<0.05 (*), p<0.01 (**), p<0.001 (***), p<0.0001 (****).

Next, we explored mammalian target of rapamycin (mTOR) pathway-dependent HIF1α protein accumulation in adherent neutrophils, as the mTOR pathway is a canonical regulator of translation in numerous cell types (41). Using phospho-specific antibodies, we found basal mTOR phosphorylation (Ser2448) in freshly isolated neutrophils adhering to FN that was augmented by GM-CSF treatment and reduced by the mTOR inhibitor rapamycin (RAP) (**Figure 6A**), indicating the RAP-mediated inhibition of mTOR autophosphorylation (42). Consequently, RAP partly reduced HIF1α protein accumulation in GM-CSF/ROX-stimulated neutrophils adherent to FN (**Figure 6B**) without affecting HIF1α transcription (**Figure 6C**). Together, these data support the notion that GM-CSF-induced HIF1α transcription and β_2_-integrin-mediated HIF1α mRNA translation as a *conditio sine qua non* cooperate in HIF1α protein upregulation. HIF1α translation but not transcription is, at least in part, controlled by the mTOR pathway.

### 3.6 HIF1α activation restriction to β_2_-integrin activation prolongs neutrophil survival

**Figure 7:**
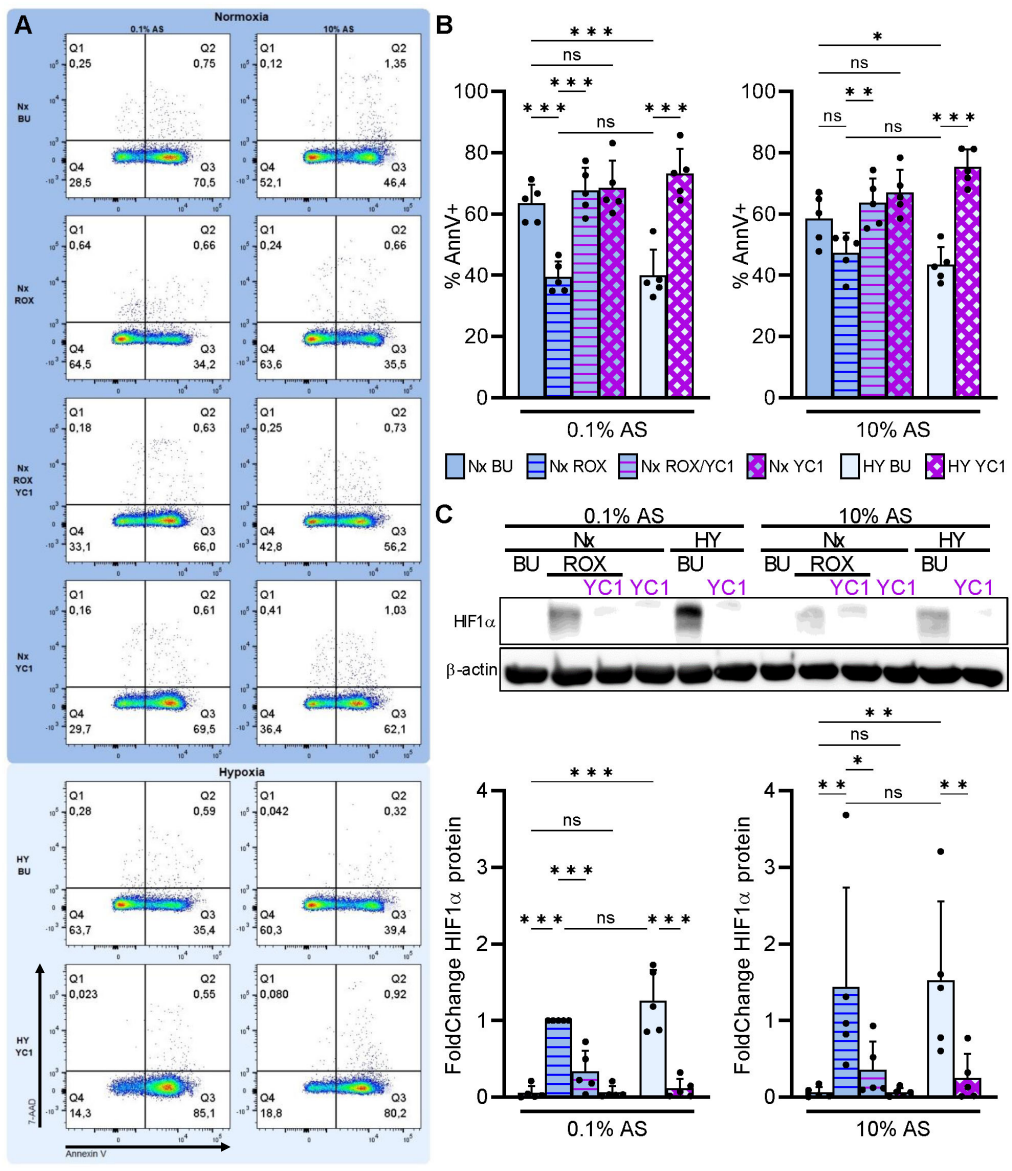
Integrin-dependent HIF1α retards neutrophil apoptosis in extravascular microenvironments. Freshly isolated human neutrophils were cultured in RPMI medium supplemented with either 0.1% or 10% autologous serum (AS) in fibronectin-coated wells (FN) under normoxia (21% O_2_, Nx, deep blue) or normobaric hypoxia (1% O_2_, HY, pale blue), and treated with 15 µM ROX, 10 µM YC1, or buffer (BU), as indicated. **(A)** Apoptosis was measured by Annexin V-APC labeling and 7-AAD staining after 20 h (n=5). Dot plots of a representative experiment are shown. **(B)** The corresponding statistics of all apoptosis measurements are given. **(C)** HIF1α immunoblotting was performed in parallel after a 4h incubation. A representative blot is depicted together with the corresponding statistics. **(B, C)** Bars of normoxic (deep blue) and hypoxic (pale blue) experimental conditions are filled with the same color as the dot plot background in **(A)**. Statistical analysis was performed by repeated measure one-way ANOVA with Šidák’s multiple comparison test. ns, not significant. p<0.05 (*), p<0.01 (**), p<0.001 (***).

Neutrophils that migrate from the circulation toward the inflammatory site leave the serum-rich blood, engage β_2_-integrins by interacting with extracellular matrix, and encounter hypoxia. We mimicked these conditions by incubating neutrophils with high (10% v/v) and low (0.1% v/v) autologous serum (AS) concentration on FN either under pseudohypoxia (ROX) or normobaric hypoxia (1% O_2_). Apoptosis was measured after 20 h in the absence or presence of the HIF1α inhibitor YC1 to determine whether HIF1α has a role in neutrophil survival under these conditions (**Figures 7A, B**). In low serum concentration (0.1% AS), we observed significantly increased HIF1α protein (**Figure 7C**) together with delayed neutrophil apoptosis under pseudohypoxia (ROX) and hypoxia. The HIF1α inhibitor YC1 not only abrogated HIF1α protein but also reversed delayed neutrophil apoptosis under pseudohypoxia and hypoxia. The apoptosis-delaying effects of pseudohypoxia and hypoxia were less pronounced in high serum concentration (10% AS) but were nevertheless counteracted by YC1. Our data suggest that HIF1α activation promotes neutrophil survival, especially after the cells left the serum-rich blood circulation migrating to their destination in the tissue.

## 4 Discussion

Neutrophils and monocytes are exposed to hypoxia during health (43) and a wide spectrum of diseases ranging from malignancies (44) to various inflammatory conditions (5, 45). During inflammation, myeloid cells employ integrins to emigrate from the blood circulation to inflammatory sites (2), becoming exposed to inflammatory mediators and hypoxia (3). Hypoxia leads to PHD inhibition, allowing HIF pathway activation. Recently, drugs have been developed that inhibit PHDs independent of oxygen tension, a condition named pseudohypoxia. Our study investigated the interplay between hypoxia or pseudohypoxia, inflammatory mediators, and adhesion in human myeloid cells and revealed several important findings. First, various inflammatory mediators did not induce HIF proteins but caused additive or synergistic HIF1α activation under hypoxic and pseudohypoxic conditions in human neutrophils, an effect not observed in human monocytes. Second, β_2_-integrins were indispensable for HIF1α protein increase restricting this pathway to neutrophils engaging β_2_-integrins. Mechanistically, continuous HIF1α transcription was necessary but not sufficient for HIF1α protein accumulation unless β_2_-integrins initiated mRNA translation. Third, HIF1α activation consistently delayed apoptosis in neutrophils interacting in low serum concentrations with extracellular matrix under hypoxia and pseudohypoxia—factors characteristic for neutrophils at extravascular inflammatory sites.

In contrast to our findings in human cells, HIF1α activation was reported in murine myeloid cells stimulated with inflammatory mediators under normoxic conditions, including LPS and bacterial components that were also used in our experiments (24, 25). Data on primary human myeloid cells are scarce (26). In agreement with these data, we found that inflammatory mediators did not induce significant HIF1α protein under normoxia. However, once human neutrophils were exposed to hypoxia or pseudohypoxia, some of these compounds provided additive or synergistic HIF1α-activating effects. Of note, despite several positive control experiments, we did not detect HIF2α protein. In contrast to human neutrophils, no additive or synergistic signal was provided by the same compounds in human monocytes. Conceivably, our observations are relevant at hypoxic inflammatory sites or when patients treated with PHD inhibitors acquire infections.

Our study expands insights into the regulation of HIF pathway activation in human neutrophils, providing a new HIF-restraining mechanism in which β_2_-integrin engagement is indispensable for HIF1α protein increase. We used fibronectin as a natural ligand (46–48) as well as activating and blocking antibodies to CD11b/CD18 to characterize this CD11b/CD18 receptor-dependent mechanism. Our experiments focused on CD11b/CD18, the most abundant β_2_-integrin (49, 50), and did not exclude similar effects of other β_2_-integrins on HIF1α protein. PECAM-1, which controls homotypic neutrophil interactions (51, 52), did not provide the necessary co-stimulus for HIF1α activation in neutrophils (**Supplementary Figure 5**). We propose that suspension neutrophils are not capable of strong HIF activation even when PHDs are inhibited or in combination with inflammatory mediators. This observation may become relevant in sepsis patients with hypoxia and in autoimmune vasculitis patients with high circulating (53) or kidney GM-CSF (54) levels. In fact, our findings are consistent with studies that failed to detect HIF signatures in human peripheral blood neutrophils (55) despite recurring hypoxia exposure in, for example, dermal, intestinal, and renal capillaries (43, 56, 57). Moreover, a recent study in patients with severe COVID-19 combined peripheral blood single-cell RNA sequencing with single-cell proteomics. Despite hypoxia, a HIF1α transcriptomic signature in one of eight identified neutrophil subclusters did not translate into proteomic data and transcription factor network analysis (27). By contrast, transcriptomic sequencing of transmigrated human neutrophils sampled from nasopharyngeal swabs (28) or broncho-alveolar fluid (29) in COVID-19 revealed upregulation of the HIF1α downstream target gene *VEGFA*, indicating successful HIF1α protein translation (28, 29). Recently, the mechanosensor PIEZO1 was implicated in neutrophil HIF1α stabilization during transmigration (58). Future studies investigating the interaction between β_2_-integrins, HIFs, and PIEZO1-mediated mechanosensation in human neutrophils are needed.

Dissecting the synergistic GM-CSF effect, we identified a strong transcriptional HIF1α response to GM-CSF stimulation via the JAK2-STAT3 pathway. Other groups also demonstrated that GM-CSF upregulated HIF1α transcription via STAT3 in myeloid cell types (59). ActD promptly shut down HIF1α protein, indicating high HIF1α mRNA turnover with a short half-life in human neutrophils. Even with PHD inhibition, continuous HIF1α transcription was necessary to ensure HIF1α protein abundance. Notably, GM-CSF strongly increased constitutive HIF1α transcription in both suspended and adherent neutrophils, yet the corresponding protein was detected only in the latter. Together, these data established that HIF1α transcription is necessary but not sufficient for HIF1α protein. Furthermore, our experiments using blocking and activating β_2_-integrin antibodies underscored that HIF1α transcription is independent from integrin signaling. Subsequent experiments revealed the indispensable mechanistic role of β_2_-integrins for the protein translation process mediated by the phosphorylation of essential initiation factors. In fact, the phosphorylation of 4EBP1 and eIF4E was shown to be essential for the initiation of 5′-cap-dependent translation (38, 60). As the mTOR pathway is a critical regulator of protein translation (41, 61, 62), we investigated the mTOR contribution to HIF1α protein accumulation in human neutrophils. We found that GM-CSF-enhanced mTOR activity in adherent neutrophils contributed to HIF1α protein accumulation. Thus, both JAK2-STAT3 and mTOR inhibition reduced GM-CSF-induced HIF1α protein accumulation, but through two distinct mechanisms. The JAK2-STAT3 pathway targeted HIF1α transcription, whereas mTOR acted via HIF1α translation. In the context of HIF activation and human neutrophil survival, we observed that HIF1α delayed constitutive apoptosis in neutrophils exposed to a typical extravascular microenvironment where β_2_-integrins engage extracellular matrix components, O_2_ tension is low, and serum factors are diluted. Thus, our data support the notion that HIF1α controls the neutrophil fate during recruitment to tissues. The importance of β_2_-integrins for the survival of migrated neutrophils is supported by a study by Haist et al. in which the investigators detected increased apoptotic rates in transmigrated CD18-defective neutrophils compared with CD18-competent neutrophils in a murine model of pulmonary infection (63). However, the specific role of HIF was not studied. Delayed human neutrophil apoptosis by HIF1α activation was reported in some (64, 65) but not all (17) previous studies. Conceivably, culturing neutrophils under strict β_2_-integrin-engaging and low serum factor conditions in our study revealed the consistent HIF1α dependency of the hypoxic and pseudohypoxic anti-apoptotic effect. Whether or not additional human neutrophil functions are HIF-controlled remains to be determined.

In summary, we describe complex interactions of hypoxic and pharmacological PHD inhibition with inflammatory mediators and β_2_-integrins cooperating in the HIF1α pathway activation in human neutrophils. We characterize a novel translation mechanism that limits HIF1α activation to neutrophils engaging β_2_-integrins. We propose that this mechanism is relevant for the cell survival of neutrophils migrating to extravascular sites, e.g., in inflammatory bowel disease, abscesses, and pyelonephritis.

## Data Availability

The original contributions presented in the study are included in the article/**Supplementary Material**. Further inquiries can be directed to the corresponding author.

## Supplementary material

Supplementary Figure 1 Exclusion of reagent toxicity.

Supplementary Figure 2 Time course of HIF1α protein expression and HIF2α protein absence.

Supplementary Figure 3 The HIF1α target gene VEGFA is not upregulated in suspended neutrophils.

Supplementary Figure 4 Monoclonal β_2_-integrin antibodies do not interfere with HIF1α transcription.

Supplementary Figure 5 A blocking monoclonal PECAM-1 antibody does not reduce HIF1α protein expression.

Supplementary Figure 6 Uncropped Immunoblots.

## References

[1] Kettritz R Choi M Rolle S Wellner M Luft FC. Integrins and cytokines activate nuclear transcription factor-κB in human neutrophils. J Biol Chem. (2004) 279:2657–65. doi: 10.1074/jbc.M309778200 14613935

[2] Ley K Laudanna C Cybulsky MI Nourshargh S. Getting to the site of inflammation: The leukocyte adhesion cascade updated. Nat Rev Immunol. (2007) 7:678–89. doi: 10.1038/nri2156 17717539

[3] Fuchs K Kuehn A Mahling M Guenthoer P Hector A Schwenck J. *In vivo* hypoxia PET imaging quantifies the severity of arthritic joint inflammation in line with overexpression of hypoxia-inducible factor and enhanced reactive oxygen species generation. J Nucl Med. (2017) 58:853–60. doi: 10.2967/jnumed.116.185934 28183987

[4] Karhausen J Haase VH Colgan SP. Inflammatory hypoxia: Role of hypoxia-inducible factor. Cell Cycle. (2005) 4:255–7. doi: 10.4161/cc.4.2.1407 15655360

[5] Kling L Schreiber A Eckardt KU Kettritz R. Hypoxia-inducible factors not only regulate but also are myeloid-cell treatment targets. J Leukoc Biol. (2021) 110:61–75. doi: 10.1002/JLB.4RI0820-535R 33070368

[6] Wang GL Semenza GL. Purification and characterization of hypoxia-inducible factor 1. J Biol Chem. (1995) 270:1230–7. doi: 10.1074/jbc.270.3.1230 7836384

[7] Hoffman E Reyes H Chu F Sander F Conley L Brooks B. Cloning of a factor required for activity of the Ah (dioxin) receptor. Science (80-). (1991) 252:954–8. doi: 10.1126/science.1852076 1852076

[8] Epstein ACR Gleadle JM McNeill LA Hewitson KS O’Rourke J Mole DR. C. elegans EGL-9 and mammalian homologs define a family of dioxygenases that regulate HIF by prolyl hydroxylation. Cell. (2001) 107:43–54. doi: 10.1016/S0092-8674(01)00507-4 11595184

[9] Bruick RK McKnight SL. A conserved family of prolyl-4-hydroxylases that modify HIF. Science. (2001) 294:1337–40. doi: 10.1126/science.1066373 11598268

[10] Yu F White SB Zhao Q Lee FS. Dynamic, site-specific interaction of hypoxia-inducible factor-1alpha with the von Hippel-Lindau tumor suppressor protein. Cancer Res. (2001) 61:4136–42. 11358837

[11] Maxwell PH Wiesener MS Chang G-W Clifford SC Vaux EC Cockman ME. The tumour suppressor protein VHL targets hypoxia-inducible factors for oxygen-dependent proteolysis. Nature. (1999) 399:271–5. doi: 10.1038/20459 10353251

[12] Huang LE Gu J Schau M Bunn HF. Regulation of hypoxia-inducible factor 1α is mediated by an O2-dependent degradation domain via the ubiquitin-proteasome pathway. Proc Natl Acad Sci U.S.A. (1998) 95:7987–92. doi: 10.1073/pnas.95.14.7987 PMC20916 9653127

[13] Appelhoffl RJ Tian YM Raval RR Turley H Harris AL Pugh CW. Differential function of the prolyl hydroxylases PHD1, PHD2, and PHD3 in the regulation of hypoxia-inducible factor. J Biol Chem. (2004) 279:38458–65. doi: 10.1074/jbc.M406026200 15247232

[14] Sonia SN George S Shahi SR Ali Z Abaza A Jamil A. An overview of safety and efficacy between hypoxia-inducible factor-prolyl-hydroxylase inhibitors and erythropoietin-stimulating agents in treating anemia in chronic kidney disease patients. Cureus. (2023) 15:e42045. doi: 10.7759/cureus.42045 37602095 PMC10436024

[15] Chen N Hao C Liu BC Lin H Wang C Xing C. Roxadustat treatment for anemia in patients undergoing long-term dialysis. N Engl J Med. (2019) 381:1011–22. doi: 10.1056/NEJMoa1901713 31340116

[16] Corcoran SE O’Neill LAJ. HIF1α and metabolic reprogramming in inflammation. J Clin Invest. (2016) 126:3699–707. doi: 10.1172/JCI84431 PMC5096812 27571407

[17] Sadiku P Willson JA Ryan EM Sammut D Coelho P Watts ER. Neutrophils fuel effective immune responses through gluconeogenesis and glycogenesis. Cell Metab. (2021) 33:411–423.e4. doi: 10.1016/j.cmet.2020.11.016 33306983 PMC7863914

[18] Shalova IN Lim JY Chittezhath M Zinkernagel AS Beasley F Hernández-Jiménez E. Human monocytes undergo functional re-programming during sepsis mediated by hypoxia-inducible factor-1α. Immunity. (2015) 42:484–98. doi: 10.1016/j.immuni.2015.02.001 25746953

[19] Thompson AAR Dickinson RS Murphy F Thomson JP Marriott HM Tavares A. Hypoxia determines survival outcomes of bacterial infection through HIF-1α–dependent reprogramming of leukocyte metabolism. Sci Immunol. (2017) 2:eaal2861. doi: 10.1126/sciimmunol.aal2861 28386604 PMC5380213

[20] Lin N Shay JES Xie H Lee DSM Skuli N Tang Q. Myeloid cell hypoxia-inducible factors promote resolution of inflammation in experimental colitis. Front Immunol. (2018) 9:2565. doi: 10.3389/fimmu.2018.02565 30455703 PMC6230677

[21] Cramer T Yamanishi Y Clausen BE Förster I Pawlinski R Mackman N. HIF-1α is essential for myeloid cell-mediated inflammation. Cell. (2003) 112:645–57. doi: 10.1016/S0092-8674(03)00154-5 PMC4480774 12628185

[22] Hua X Hu G Hu Q Chang Y Hu Y Gao L. Single-cell RNA sequencing to dissect the immunological network of autoimmune myocarditis. Circulation. (2020) 142:384–400. doi: 10.1161/CIRCULATIONAHA.119.043545 32431172

[23] Sadiku P Willson JA Dickinson RS Murphy F Harris AJ Lewis A. Prolyl hydroxylase 2 inactivation enhances glycogen storage and promotes excessive neutrophilic responses. J Clin Invest. (2017) 127:3407–20. doi: 10.1172/JCI90848 PMC5669581 28805660

[24] Peyssonnaux C Datta V Cramer T Doedens A Theodorakis EA Gallo RL. HIF-1alpha expression regulates the bactericidal capacity of phagocytes. J Clin Invest. (2005) 115:1806–15. doi: 10.1172/JCI23865 PMC1159132 16007254

[25] Peyssonnaux C Cejudo-Martin P Doedens A Zinkernagel AS Johnson RS Nizet V. Cutting edge: Essential role of hypoxia inducible factor-1alpha in development of lipopolysaccharide-induced sepsis. J Immunol. (2007) 178:7516–9. doi: 10.4049/jimmunol.178.12.7516 17548584

[26] Thompson AAR Elks PM Marriott HM Eamsamarng S Higgins KR Lewis A. Hypoxia-inducible factor 2a regulates key neutrophil functions in humans, mice, and zebrafish. Blood. (2014) 123:366–76. doi: 10.1182/blood-2013-05-500207 PMC3894493 24196071

[27] Schulte-Schrepping J Reusch N Paclik D Baßler K Schlickeiser S Zhang B. Severe COVID-19 is marked by a dysregulated myeloid cell compartment. Cell. (2020) 182:1419–1440.e23. doi: 10.1016/j.cell.2020.08.001 32810438 PMC7405822

[28] Trump S Lukassen S Anker MS Chua RL Liebig J Thürmann L. Hypertension delays viral clearance and exacerbates airway hyperinflammation in patients with COVID-19. Nat Biotechnol. (2021) 39:705–16. doi: 10.1038/s41587-020-00796-1 33361824

[29] Silvin A Chapuis N Dunsmore G Goubet A-G Dubuisson A Derosa L. Elevated calprotectin and abnormal myeloid cell subsets discriminate severe from mild COVID-19. Cell. (2020) 182:1401–1418.e18. doi: 10.1016/j.cell.2020.08.002 32810439 PMC7405878

[30] Schreiber A Busjahn A Luft FC Kettritz R. Membrane expression of proteinase 3 is genetically determined. J Am Soc Nephrol. (2003) 14:68–75. doi: 10.1097/01.ASN.0000040751.83734.D1 12506139

[31] Schreiber A Luft FC Kettritz R. Phagocyte NADPH oxidase restrains the inflammasome in ANCA-induced GN. J Am Soc Nephrol. (2015) 26:411–24. doi: 10.1681/ASN.2013111177 PMC4310655 25012177

[32] Zhang Y Jing Y Zhou C. Correlation between blood concentration of roxadustat and clinical efficacy in patients with anemia of chronic kidney disease. Med (Baltimore). (2023) 102:e33564. doi: 10.1097/MD.0000000000033564 PMC10101297 37058012

[33] Groenendaal-van de Meent D Kerbusch V Kaspera R Barroso-Fernandez B Galletti P Klein GK. Effect of kidney function and dialysis on the pharmacokinetics and pharmacodynamics of roxadustat, an oral hypoxia-inducible factor prolyl hydroxylase inhibitor. Eur J Drug Metab Pharmacokinet. (2021) 46:141–53. doi: 10.1007/s13318-020-00658-w PMC7811989 33165773

[34] Takada A Shibata T Shiga T Groenendaal-van de Meent D Komatsu K. Population pharmacokinetics of roxadustat in Japanese dialysis-dependent chronic kidney disease patients with anaemia. Br J Clin Pharmacol. (2022) 88:787–97. doi: 10.1111/bcp.15023 PMC9292185 34350625

[35] Kamoshida G Kikuchi-Ueda T Nishida S Tansho-Nagakawa S Ubagai T Ono Y. Pathogenic bacterium Acinetobacter baumannii inhibits the formation of neutrophil extracellular traps by suppressing neutrophil adhesion. Front Immunol. (2018) 9:178. doi: 10.3389/fimmu.2018.00178 29467765 PMC5808340

[36] Liu X Chen H Patel DJ. Solution structure of actinomycin-DNA complexes: drug intercalation at isolated G-C sites. J Biomol NMR. (1991) 1:323–47. doi: 10.1007/BF02192858 1841703

[37] Schneider-Poetsch T Ju J Eyler DE Dang Y Bhat S Merrick WC. Inhibition of eukaryotic translation elongation by cycloheximide and lactimidomycin. Nat Chem Biol. (2010) 6:209–17. doi: 10.1038/nchembio.304 PMC2831214 20118940

[38] Sonenberg N Hinnebusch AG. Regulation of translation initiation in eukaryotes: mechanisms and biological targets. Cell. (2009) 136:731–45. doi: 10.1016/j.cell.2009.01.042 PMC3610329 19239892

[39] Moerke NJ Aktas H Chen H Cantel S Reibarkh MY Fahmy A. Small-Molecule Inhibition of the Interaction between the Translation Initiation Factors eIF4E and eIF4G. Cell. (2007) 128:257–67. doi: 10.1016/j.cell.2006.11.046 17254965

[40] Lucchesi CA Zhang J Gao M Shaw J Chen X. Identification of a first-in-class small-molecule inhibitor of the EIF4E-RBM38 complex that enhances wild-type TP53 protein translation for tumor growth suppression. Mol Cancer Ther. (2023) 22:726–36. doi: 10.1158/1535-7163.MCT-22-0627 PMC10866396 36940176

[41] Brunn GJ Hudson CC Sekulić A Williams JM Hosoi H Houghton PJ. Phosphorylation of the translational repressor PHAS-I by the mammalian target of rapamycin. Science (80-). (1997) 277:99–101. doi: 10.1126/science.277.5322.99 9204908

[42] Zhang X Mu X Huang O Xie Z Jiang M Geng M. Luminal breast cancer cell lines overexpressing ZNF703 are resistant to tamoxifen through activation of akt/mTOR signaling. PloS One. (2013) 8:1–10. doi: 10.1371/journal.pone.0072053 PMC3753350 23991038

[43] Ortiz-Prado E Dunn JF Vasconez J Castillo D. Viscor G. Partial pressure of oxygen in the human body: a general review. Am J Blood Res. (2019) 9:1–14. 30899601 PMC6420699

[44] Mahiddine K Blaisdell A Ma S Créquer-Grandhomme A Lowell CA Erlebacher A. Relief of tumor hypoxia unleashes the tumoricidal potential of neutrophils. J Clin Invest. (2020) 130:389–403. doi: 10.1172/JCI130952 31600172 PMC6934192

[45] Chen PM Wilson PC Shyer JA Veselits M Steach HR Cui C. Kidney tissue hypoxia dictates T cell–mediated injury in murine lupus nephritis. Sci Transl Med. (2020) 12. doi: 10.1126/scitranslmed.aay1620 PMC8055156 32269165

[46] Owen CA Campbell EJ Stockley RA. Monocyte adherence to fibronectin: role of CD11/CD18 integrins and relationship to other monocyte functions. J Leukoc Biol. (1992) 51:400–8. doi: 10.1002/jlb.51.4.400 1348780

[47] van den Berg JM Mul FP Schippers E Weening JJ Roos D Kuijpers TW. Beta1 integrin activation on human neutrophils promotes beta2 integrin-mediated adhesion to fibronectin. Eur J Immunol. (2001) 31:276–84. doi: 10.1002/1521-4141(200101)31:1<276::AID-IMMU276>3.0.CO;2-D 11265644

[48] Lamers C Plüss CJ Ricklin D. The promiscuous profile of complement receptor 3 in ligand binding, immune modulation, and pathophysiology. Front Immunol. (2021) 12:662164. doi: 10.3389/fimmu.2021.662164 33995387 PMC8118671

[49] Bouti P Webbers SDS Fagerholm SC Alon R Moser M Matlung HL. [amp]]beta;2 integrin signaling cascade in neutrophils: more than a single function. Front Immunol. (2020) 11:619925. doi: 10.3389/fimmu.2020.619925 33679708 PMC7930317

[50] Lakschevitz FS Hassanpour S Rubin A Fine N Sun C Glogauer M. Identification of neutrophil surface marker changes in health and inflammation using high-throughput screening flow cytometry. Exp Cell Res. (2016) 342:200–9. doi: 10.1016/j.yexcr.2016.03.007 26970376

[51] Dangerfield J Larbi KY Huang M-T Dewar A Nourshargh S. PECAM-1 (CD31) homophilic interaction up-regulates alpha6beta1 on transmigrated neutrophils in *vivo* and plays a functional role in the ability of alpha6 integrins to mediate leukocyte migration through the perivascular basement membrane. J Exp Med. (2002) 196:1201–11. doi: 10.1084/jem.20020324 PMC2194111 12417630

[52] Christofidou-Solomidou M Nakada MT Williams J Muller WA DeLisser HM. Neutrophil platelet endothelial cell adhesion molecule-1 participates in neutrophil recruitment at inflammatory sites and is down-regulated after leukocyte extravasation. J Immunol. (1997) 158:4872–8. doi: 10.4049/jimmunol.158.10.4872 9144503

[53] Presneill JJ Harley NS Wilson JW Cade JF Waring PM Layton JE. Plasma granulocyte colony-stimulating factor and granulocyte-macrophage colony-stimulating factor levels in critical illness including sepsis and septic shock: Relation to disease severity, multiple organ dysfunction, and mortality. Crit Care Med. (2000) 28:2344–54. doi: 10.1097/00003246-200007000-00028 10921563

[54] Rousselle A Sonnemann J Amann K Mildner A Lodka D Kling L. CSF2-dependent monocyte education in the pathogenesis of ANCA-induced glomerulonephritis. Ann Rheum Dis. (2022) 81:1162–72. doi: 10.1136/annrheumdis-2021-221984 PMC9279749 35418479

[55] Jun HS Weinstein DA Lee YM Mansfield BC Chou JY. Molecular mechanisms of neutrophil dysfunction in glycogen storage disease type Ib. Blood. (2014) 123:2843–53. doi: 10.1182/blood-2013-05-502435 PMC4007611 24565827

[56] Epstein FH Agmon Y Brezis M. Physiology of renal hypoxia. Ann N Y Acad Sci. (1994) 718:72–81;discussion 81-2. doi: 10.1111/j.1749-6632.1994.tb55706.x 8185253

[57] Kelly CJ Zheng L Campbell EL Saeedi B Scholz CC Bayless AJ. Crosstalk between microbiota-derived short-chain fatty acids and intestinal epithelial HIF augments tissue barrier function. Cell Host Microbe. (2015) 17:662–71. doi: 10.1016/j.chom.2015.03.005 PMC4433427 25865369

[58] Mukhopadhyay A Tsukasaki Y Chan WC Le JP Kwok ML Zhou J. trans-Endothelial neutrophil migration activates bactericidal function via Piezo1 mechanosensing. Immunity. (2024) 57:52–67.e10. doi: 10.1016/j.immuni.2023.11.007 38091995 PMC10872880

[59] Thorn M Guha P Cunetta M Espat NJ Miller G Junghans RP. Tumor-associated GM-CSF overexpression induces immunoinhibitory molecules via STAT3 in myeloid-suppressor cells infiltrating liver metastases. Cancer Gene Ther. (2016) 23:188–98. doi: 10.1038/cgt.2016.19 27199222

[60] Gingras AC Gygi SP Raught B Polakiewicz RD Abraham RT Hoekstra MF. Regulation of 4E-BP1 phosphorylation: a novel two-step mechanism. Genes Dev. (1999) 13:1422–37. doi: 10.1101/gad.13.11.1422 PMC316780 10364159

[61] Fingar DC Salama S Tsou C Harlow E Blenis J. Mammalian cell size is controlled by mTOR and its downstream targets S6K1 and 4EBP1/eIF4E. Genes Dev. (2002) 16:1472–87. doi: 10.1101/gad.995802 PMC186342 12080086

[62] Harada H Itasaka S Kizaka-Kondoh S Shibuya K Morinibu A Shinomiya K. The Akt/mTOR pathway assures the synthesis of HIF-1alpha protein in a glucose- and reoxygenation-dependent manner in irradiated tumors. J Biol Chem. (2009) 284:5332–42. doi: 10.1074/jbc.M806653200 19098000

[63] Haist M Ries F Gunzer M Bednarczyk M Siegel E Kuske M. Neutrophil-specific knockdown of β2 integrins impairs antifungal effector functions and aggravates the course of invasive pulmonal aspergillosis. Front Immunol. (2022) 13:823121. doi: 10.3389/fimmu.2022.823121 35734179 PMC9207500

[64] Walmsley SR Print C Farahi N Peyssonnaux C Johnson RS Cramer T. Hypoxia-induced neutrophil survival is mediated by HIF-1α-dependent NF-κB activity. J Exp Med. (2005) 201:105–15. doi: 10.1084/jem.20040624 PMC2212759 15630139

[65] Hannah S Mecklenburgh K Rahman I Bellingan GJ Greening A Haslett C. Hypoxia prolongs neutrophil survival in *vitro* . FEBS Lett. (1995) 372:233–7. doi: 10.1016/0014-5793(95)00986-J 7556675

