# Supplementary material for: β2-integrins control HIF1α activation in human neutrophils
Source: Front Immunol. 2024 Oct 14;15:1406967. doi: 10.3389/fimmu.2024.1406967 (PMC11513320; doi:10.3389/fimmu.2024.1406967)

Supplementary Material - Table of Contents

Supplementary Figure 1	Exclusion of reagent toxicity.	P. 2
Supplementary Figure 2	Time course of HIF1α protein expression and HIF2α absence.	P. 3
Supplementary Figure 3	The HIF1α target gene VEGFA is not upregulated in suspended neutrophils.	P. 4
Supplementary Figure 4	Monoclonal β_2-integrin antibodies do not interfere with HIF1α transcription.	P. 5
Supplementary Figure 5	A blocking monoclonal PECAM-1 antibody does not reduce HIF1α protein expression.	P. 6
Supplementary Figure 6	Uncropped Immunoblots.	P. 7-14

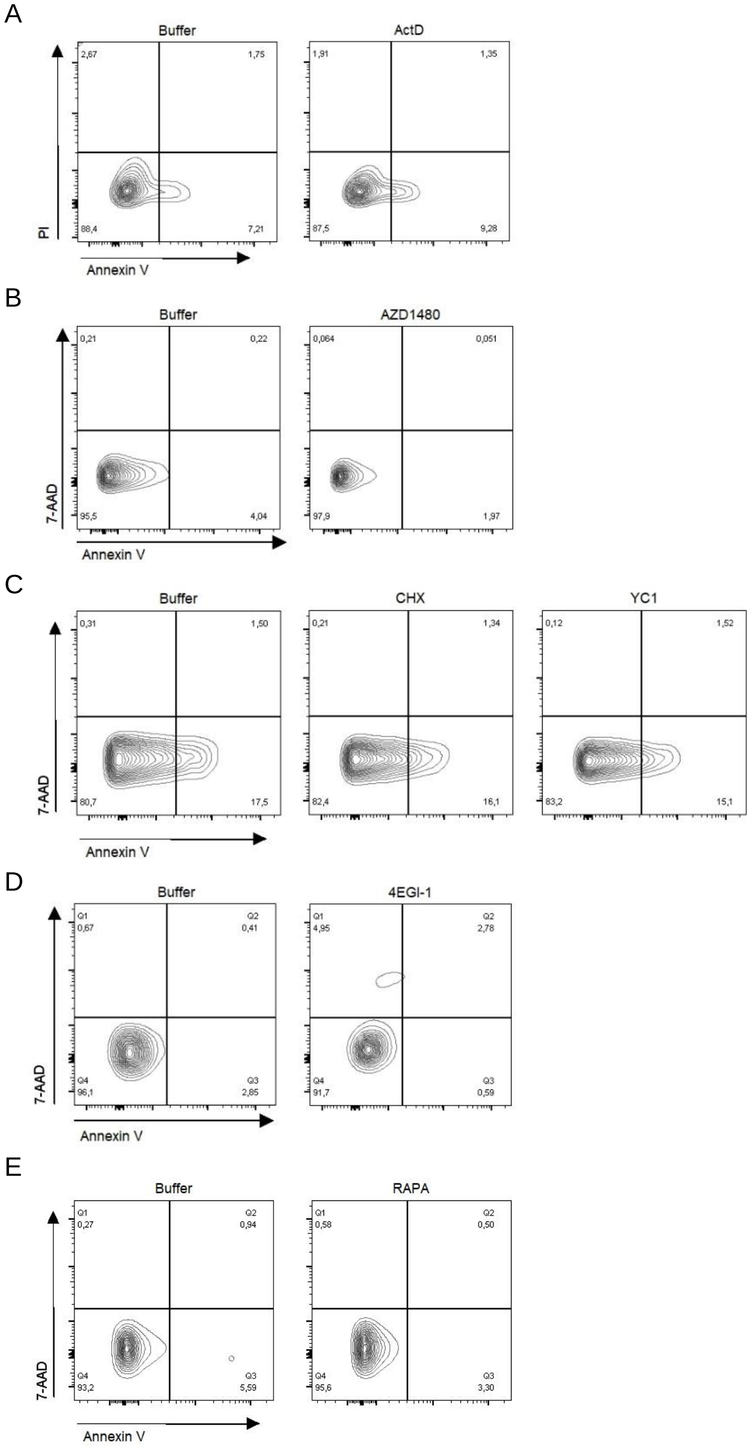

Supplementary Figure 1. Exclusion of reagent toxicity.

Using freshly isolated human neutrophils, no increased cytotoxicity was observed within 4h as compared to the appropriate buffer control with regards to 5 µg/ml Actinomycin D (ActD) (**A**), 1µM AZD1480 (**B**), 2.5µg/ml cycloheximide (CHX), 10µM YC1 (**C**), 25µM 4EGI-1 (**D**), and 100nM rapamycin (RAPA) (**D**). Note that we used propidium iodide (PI) to stain necrotic neutrophils in (**A**) as 7-AAD measurement is imprecise due to remarkable spectral overlap with ActD emitted fluorescence.

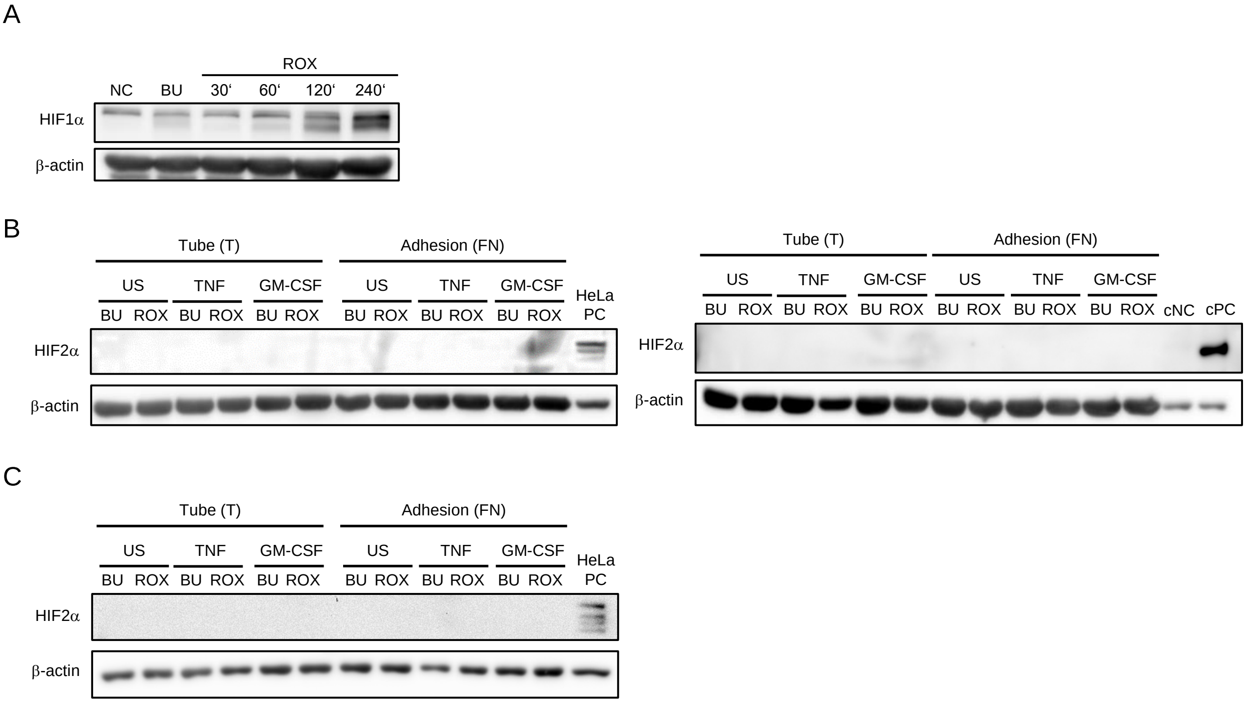

Supplementary Figure 2. Time course of HIF1α protein expression and HIF2α protein absence.

(**A**) Freshly isolated human neutrophils were treated with buffer (BU) or 15µM roxadustat (ROX) for the indicated time points prior to immunoblotting. A representative blot is shown.
(**B**) Representative HIF2α immunoblots of freshly isolated human neutrophils (**B**) and monocytes (**C**) stimulated with 2ng/ml TNFα, 20ng/ml GM-CSF or without (US) in the absence (BU) or presence of 15µM ROX for 4h are demonstrated. Antibodies in experiments to detect HIF2α were as follows: clone ep190b (1:500, Novus Biologicals), rabbit polyclonal NB100-122 (1:500, Novus Biologicals), and rabbit polyclonal PA1-16510 (1:500, Thermo Fisher Scientific, Waltham, USA). Commercial negative (cNC) and positive lysates (cPC, $CoCl_2$-treated HepG2 cells) were purchased from Cell Signaling Technology (#94790, Leiden, The Netherlands). Our own positive control (HeLa PC) was prepared by 100ng/ml IFNα and 15µM ROX treatment of HeLa cells for 2h prior to protein isolation in accordance with the protocol used for myeloid cells.

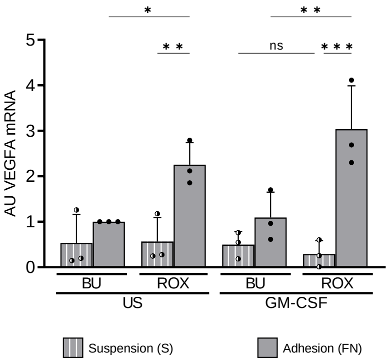

Supplementary Figure 3. The HIF1α target gene VEGFA is not upregulated in suspended neutrophils.

Freshly isolated human neutrophils were cultured on PolyHema-coated wells (hatched bars, Suspension (S)) or fibronectin(FN)-coated wells (gray bars, Adhesion (FN)) for 4h at 37°C, 5% CO_2 and treated with buffer (BU), 15µM roxadustat (ROX), 20ng/ml GM-CSF, and combinations thereof as indicated. Total RNA was isolated and mRNA expression of HIF1α target gene VEGFA (was analyzed by qPCR. The BU condition in adherent neutrophils was set as reference. Statistical analysis was performed by repeated-measure one-way ANOVA with Šidák's multiple comparison test.

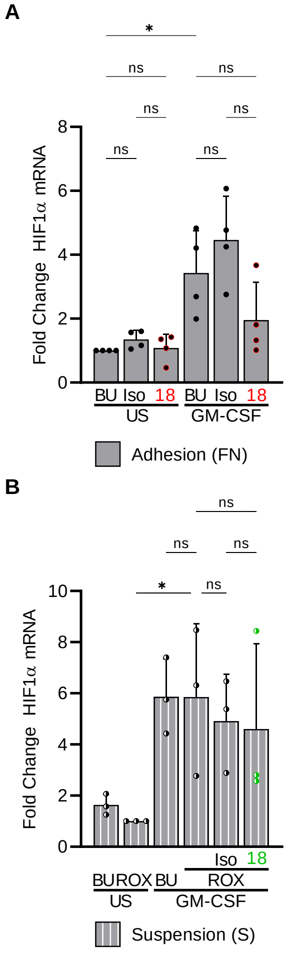

Supplementary Figure 4. Monoclonal β_2-integrin antibodies do not interfere with HIF1α transcription.

(**A**) Freshly isolated human neutrophils were pre-incubated with 20µg of blocking monoclonal CD18 antibody (18) or isotype (Iso) for 30min on ice prior to 4h incubation on fibronectin(FN)-coated wells at 37°C with buffer (BU), 20ng/ml GM-CSF or without (US), or combinations thereof as indicated. Neutrophils were prepared for HIF1α qPCR. The unstimulated BU condition was set as reference.

(**B**) Freshly isolated human neutrophils were pre-incubated with 10µg of activating monoclonal CD18 antibody (18) or isotype (Iso) for 30min on ice prior to 4h incubation on PolyHema-coated wells (Suspension (S)) at 37°C with BU, 15µM roxadustat (ROX), 20ng/ml GM-CSF, or combinations thereof as indicated. Neutrophils were prepared for HIF1α qPCR. The ROX condition was set as reference. Statistical analysis was performed by repeated-measure one-way ANOVA with Šidák's multiple comparison test.

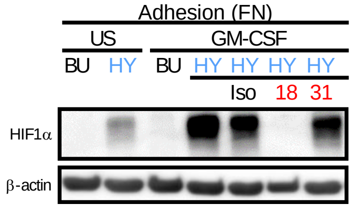

Supplementary Figure 5. A blocking monoclonal PECAM-1 antibody does not reduce HIF1α protein expression.

Freshly isolated human neutrophils were pre-incubated with 20µg of blocking monoclonal CD18 antibody (18), blocking monoclonal CD31 antibody (31) or isotype (Iso) for 30min on ice prior to 4h incubation on fibronectin(FN)-coated wells at 37°C in normobaric hypoxia (1% O_2, HY) with buffer (BU), 20ng/ml GM-CSF or without (US), or combinations thereof as indicated. Neutrophils were prepared for HIF1α immunoblot. A representative experiment of n=2 is depicted.

Supplementary Figure 6. Uncropped Immunoblots.

Figure 1A

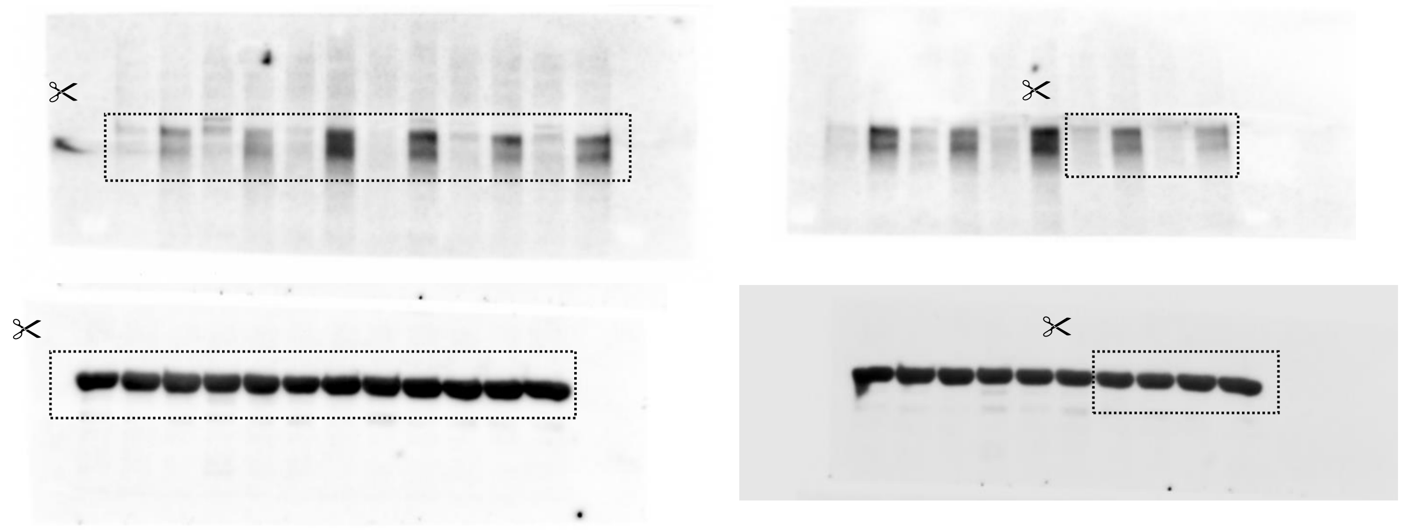

Figure 1B

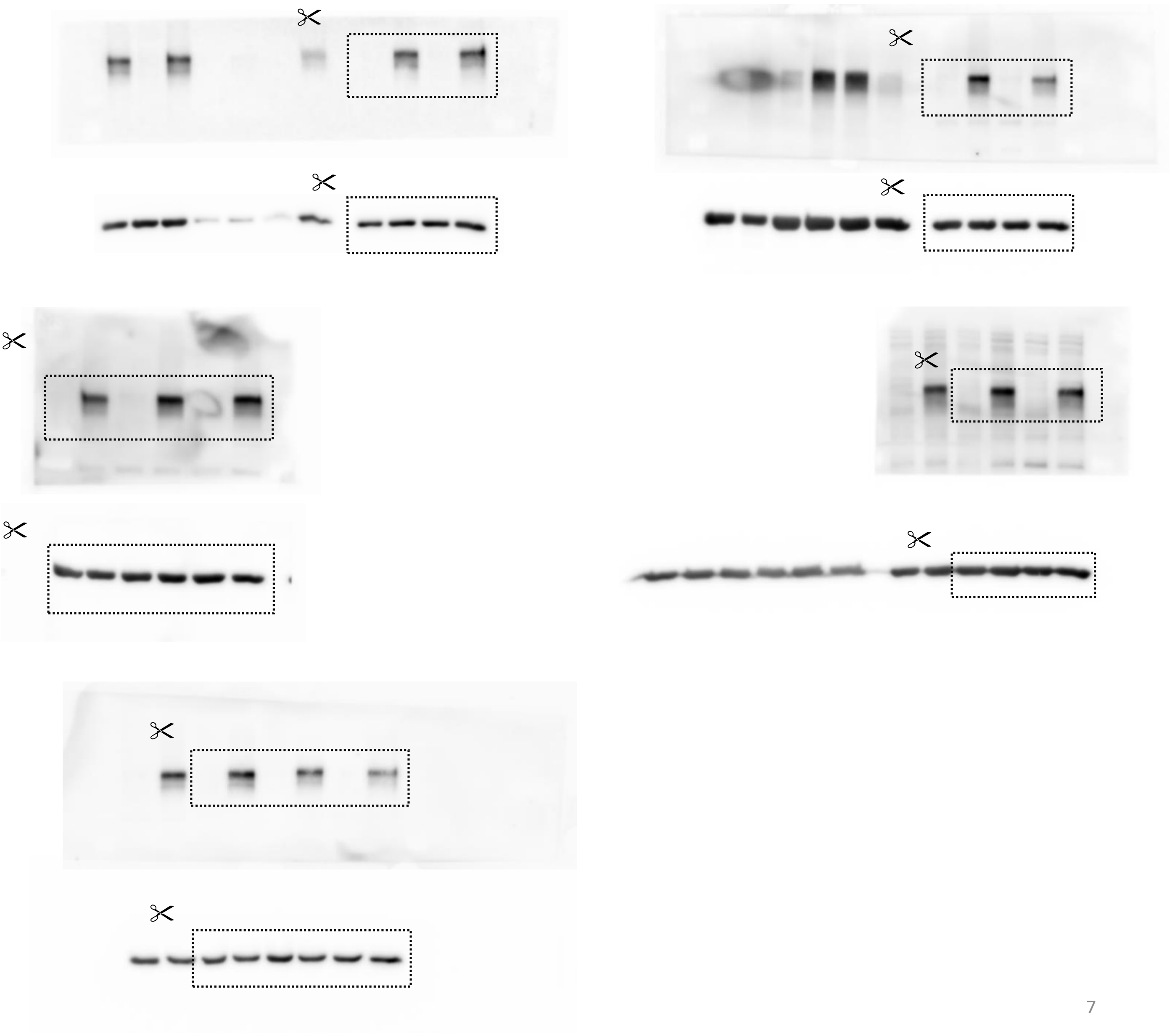

Figure 2A

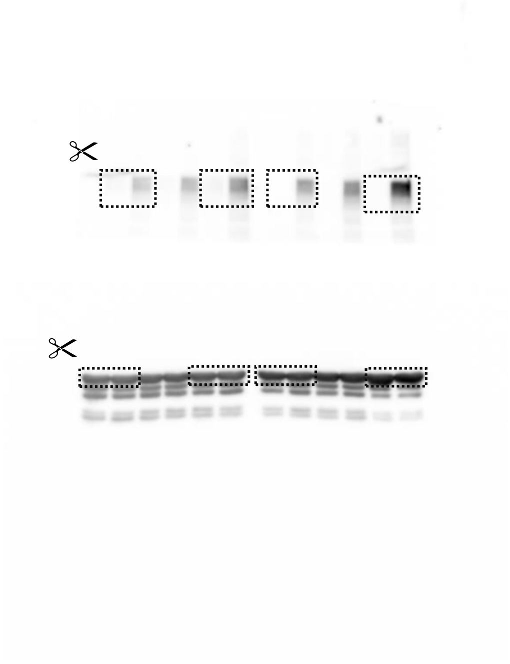

Figure 2B

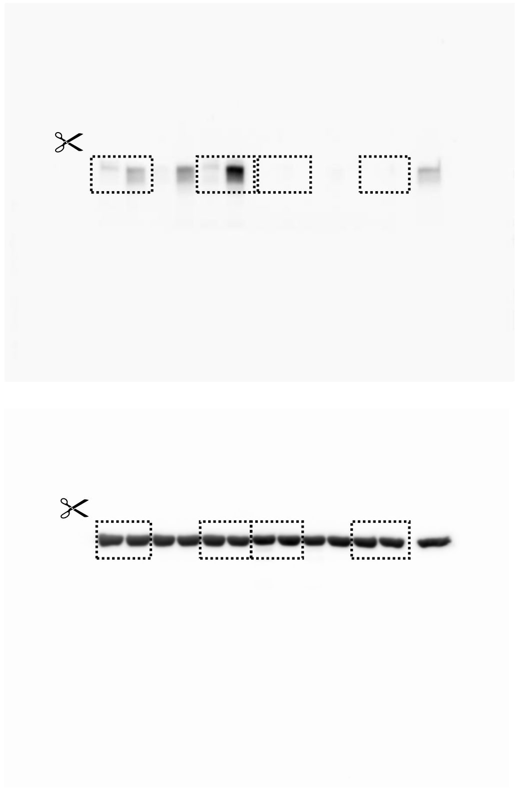

Figure 2C

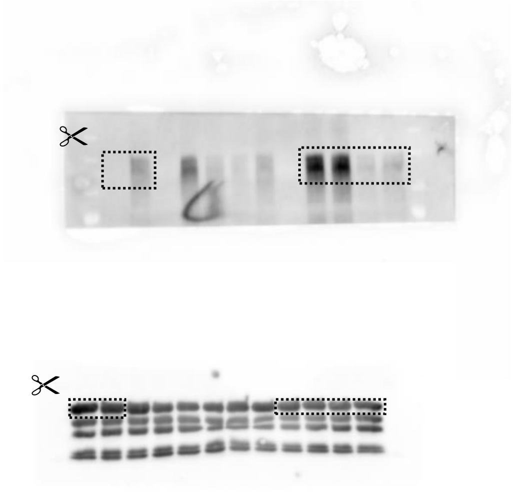

Figure 2D

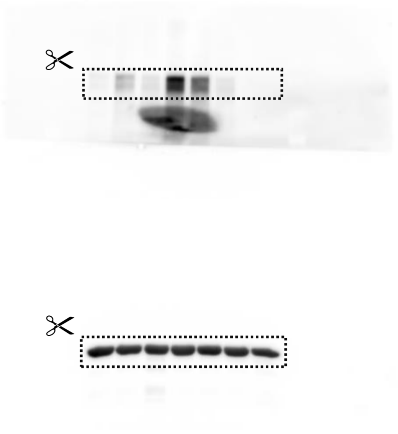

Figure 2E

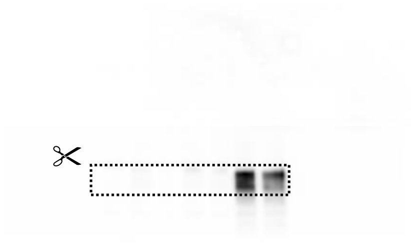

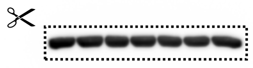

Figure 2F

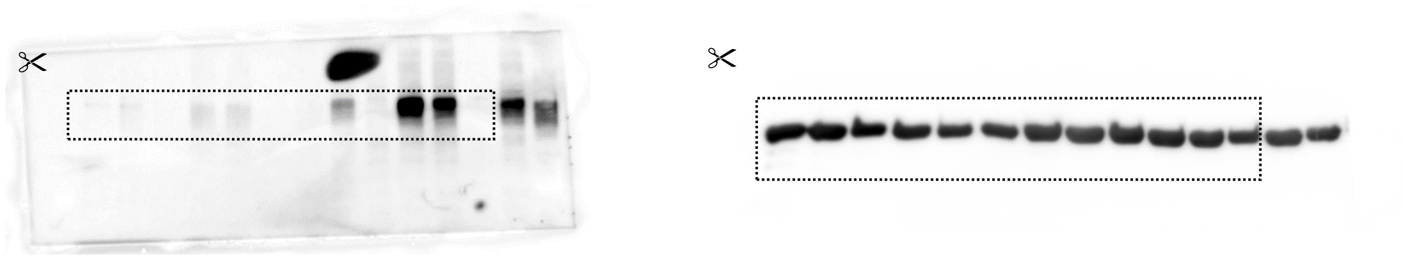

Figure 2G

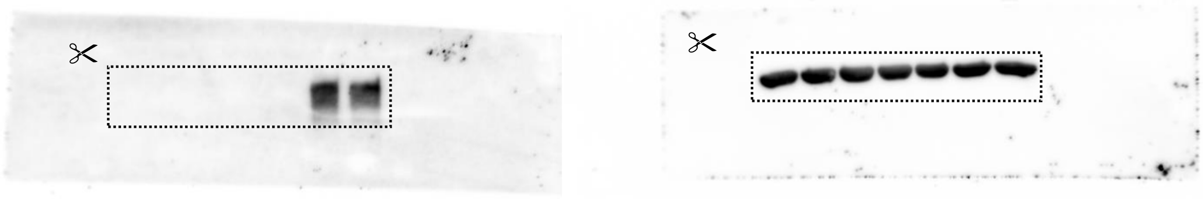

Figure 3C

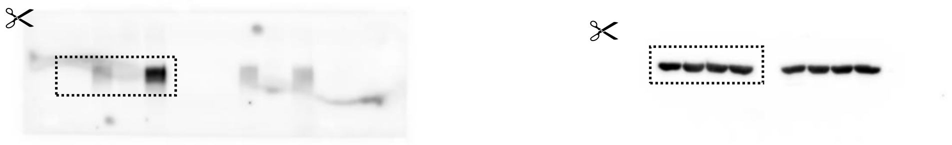

Figure 3F

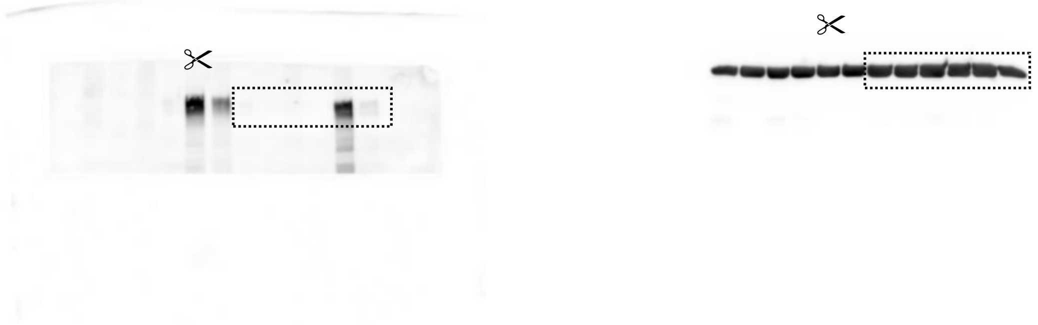

Figure 4B

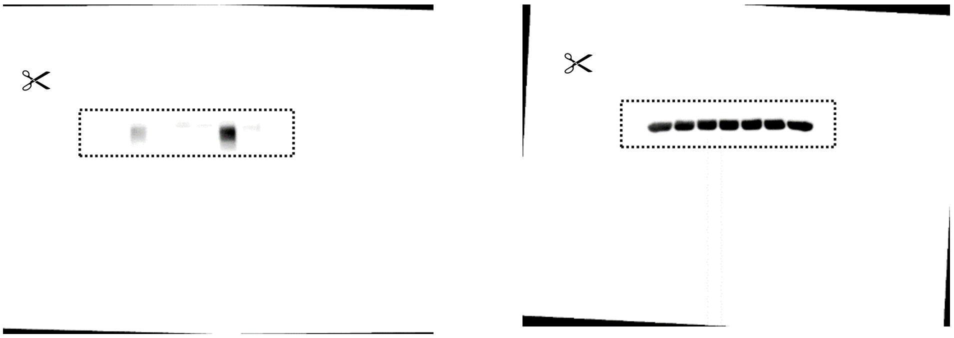

Figure 4D

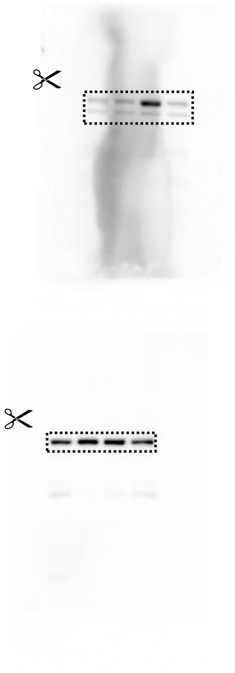

Figure 4E

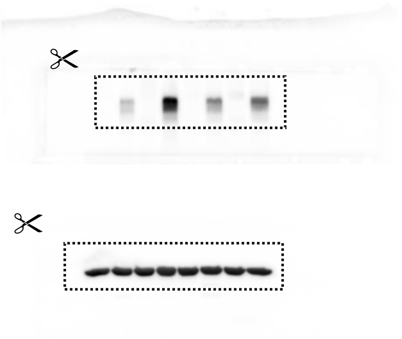

Figure 5A

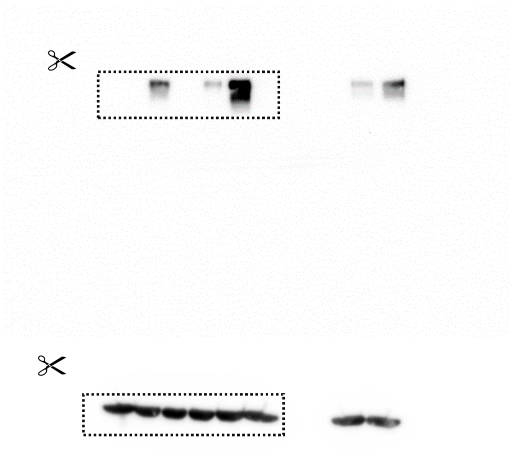

Figure 5B

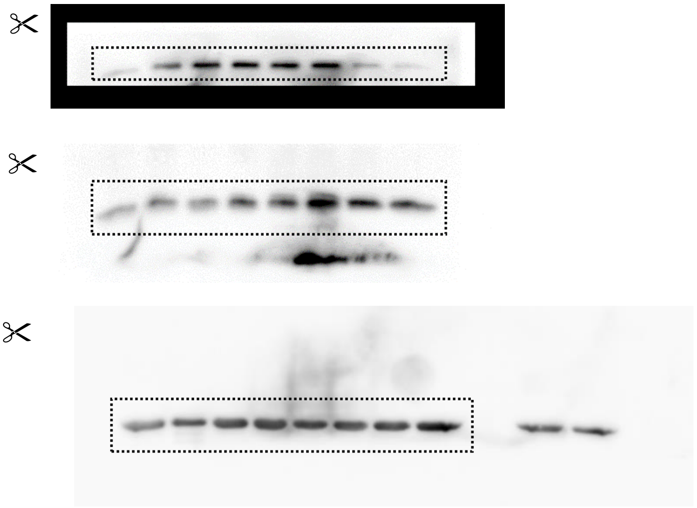

Figure 5C

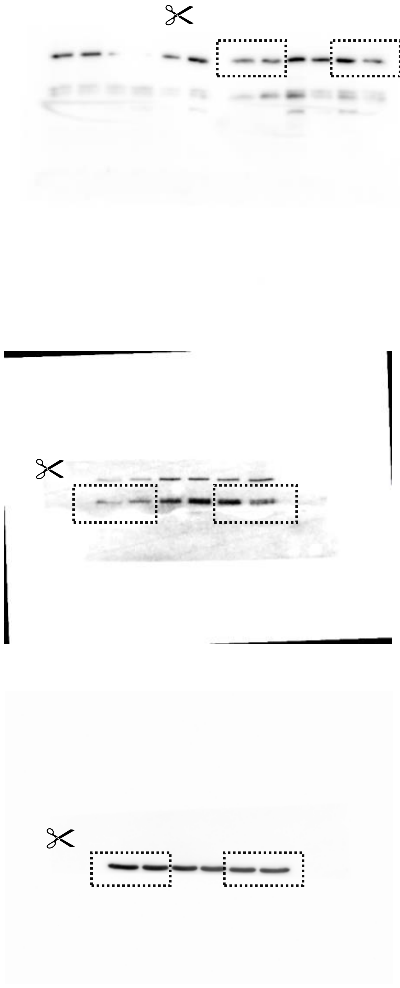

Figure 5D

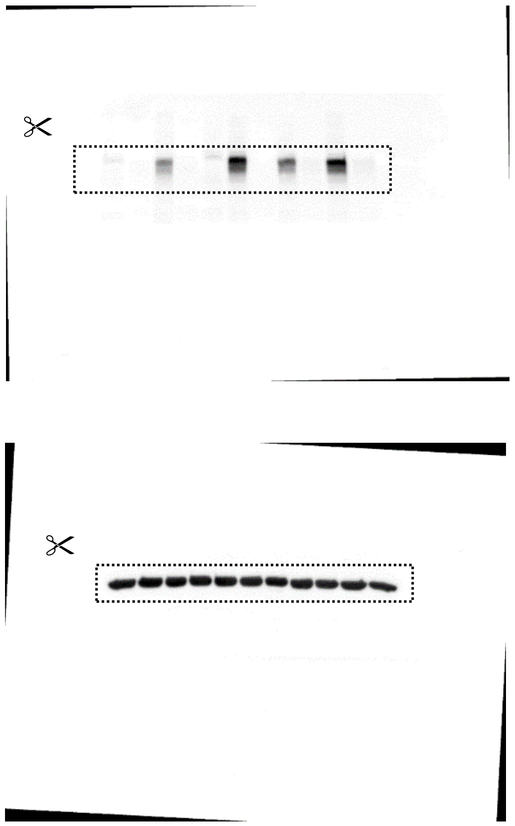

Figure 6A

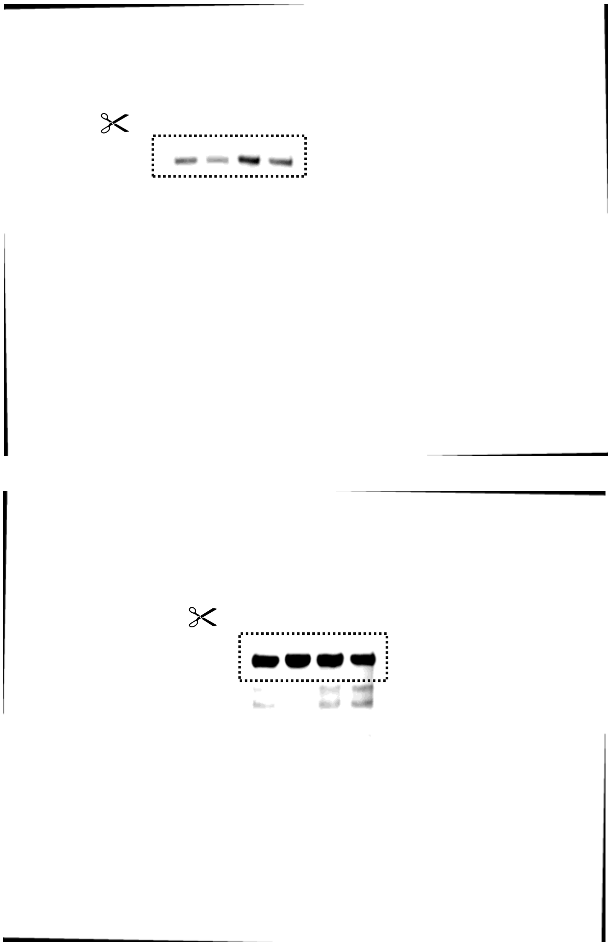

Figure 6B

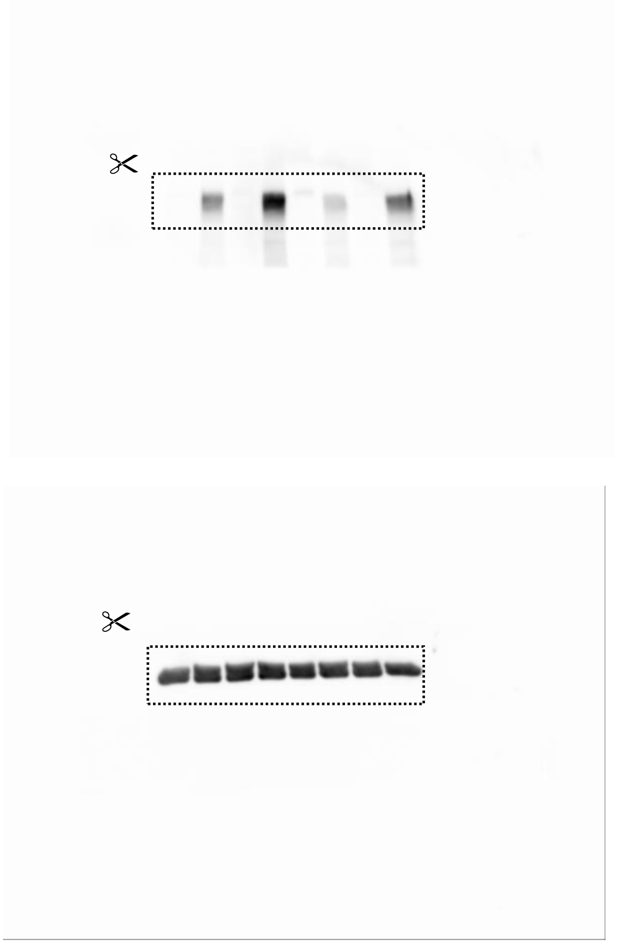

Figure 7C

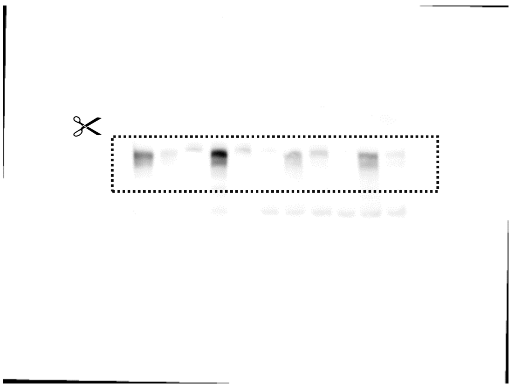

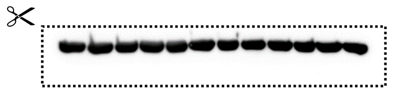

Supplementary Figure 2A

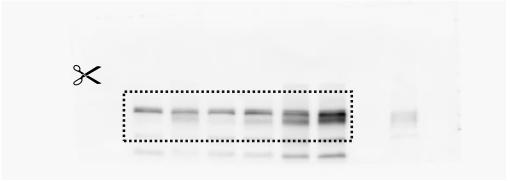

Supplementary Figure 2B

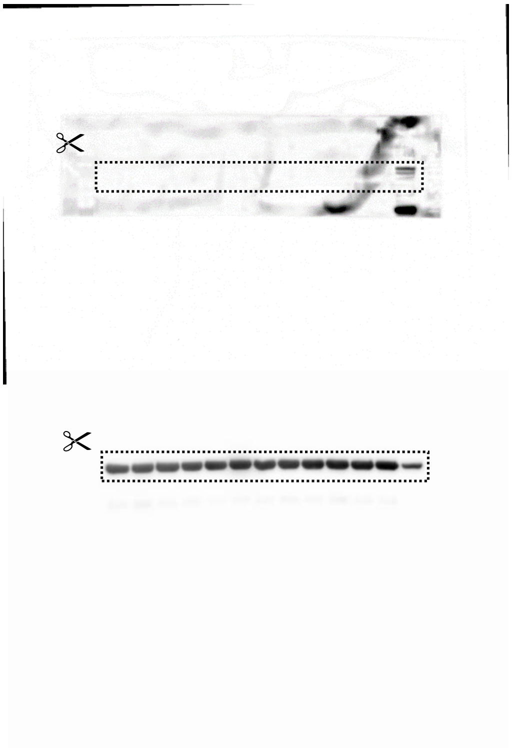

Supplementary Figure 2C

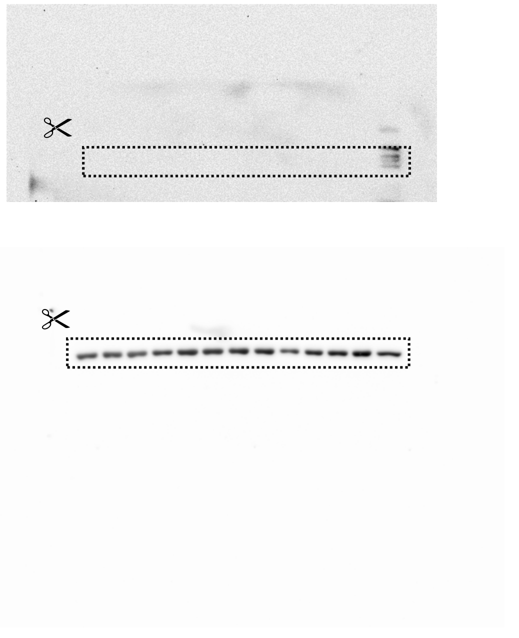

Supplementary Figure 2B

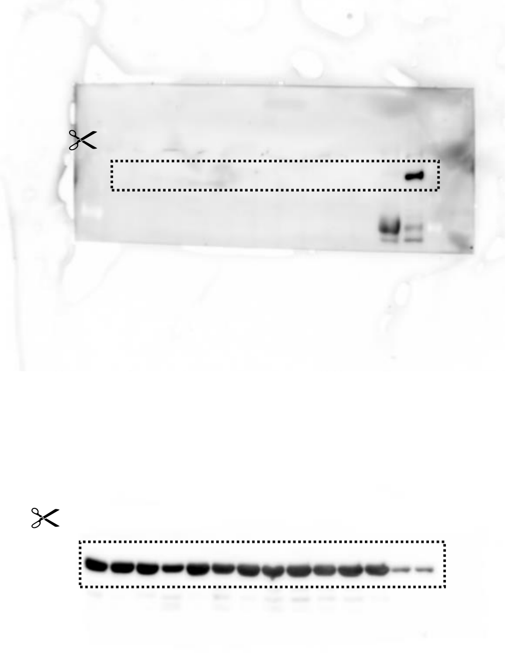

Supplementary Figure 5

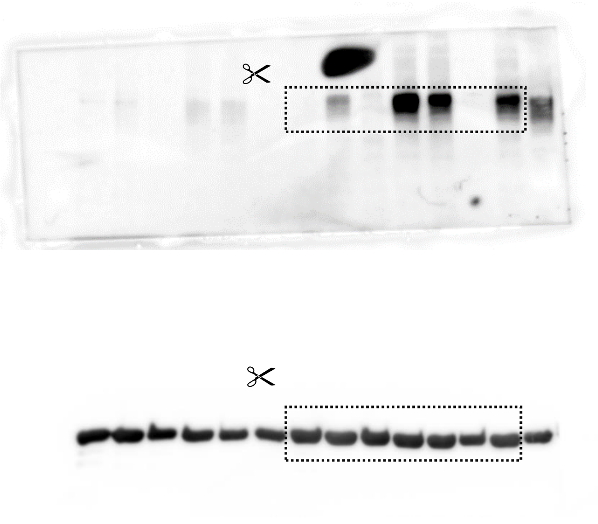

Supplement: Supplementary Figure 1 — Exclusion of reagent toxicity. [file DataSheet1.pdf]
